# Pericyte hypoxia-inducible factor-1 (HIF-1) drives blood-brain barrier disruption and impacts acute ischemic stroke outcome

**DOI:** 10.1007/s10456-021-09796-4

**Published:** 2021-05-27

**Authors:** Chih-Chieh Tsao, Julia Baumann, Sheng-Fu Huang, Diana Kindler, Aileen Schroeter, Nicole Kachappilly, Max Gassmann, Markus Rudin, Omolara O. Ogunshola

**Affiliations:** 1 Institute of Veterinary Physiology and Zurich Center for Integrative Human Physiology (ZIHP), University of Zurich, Winterthurerstrasse 260, 8057 Zurich, Switzerland; 2 Institute for Biomedical Engineering, ETH Zurich, Wolfgang-Pauli-Strasse 27, 8093 Zurich, Switzerland

**Keywords:** Cerebral ischemia, Vascular permeability, Pericyte coverage, Pericyte death

## Abstract

Pericytes play essential roles in blood-brain barrier integrity and their dysfunction is implicated in neurological disorders such as stroke although the underlying mechanisms remain unknown. Hypoxia-inducible factor-1 (HIF-1), a master regulator of injury responses, has divergent roles in different cells especially during stress scenarios. On one hand HIF-1 is neuroprotective but on the other it induces vascular permeability. Since pericytes are critical for barrier stability, we asked if pericyte HIF-1 signaling impacts barrier integrity and injury severity in a mouse model of ischemic stroke. We show that pericyte HIF-1 loss of function (LoF) diminishes ischemic damage and barrier permeability at 3 days reperfusion. HIF-1 deficiency preserved barrier integrity by reducing pericyte death thereby maintaining vessel coverage and junctional protein organization, and suppressing vascular remodeling. Importantly, considerable improvements in sensorimotor function were observed in HIF-1 LoF mice indicating that better vascular functionality post stroke improves outcome. Thus, boosting vascular integrity by inhibiting pericytic HIF-1 activation and/or increasing pericyte survival may be a lucrative option to accelerate recovery after severe brain injury.

**Supplementary Information:**

The online version contains supplementary material available at 10.1007/s10456-021-09796-4.

## Introduction

Vascular changes are much more instrumental for brain recovery after injury than previously appreciated. Indeed, brain vascular dysfunction characterizes many CNS diseases [1, 2]. The brain vasculature together with neurons, pericytes, astrocytes and microglia form a complex, yet dynamic functional network called the neurovascular unit (NVU) [3]. At the capillary level, the blood-brain barrier (BBB) compartment plays a critical role in transporting nutrients from blood to brain, preventing substances circulating in the bloodstream from gaining access to the brain parenchyma and removing toxins. Thus, the BBB maintains brain homeostasis and facilitates proper neuronal function [2, 4]. Cerebral endothelial cells are the foundation of the BBB and perform a “gate-keeper” function due to high expression of restrictive tight and adherens junction proteins and various transporters [4]. Perivascular pericytes and astrocytes, support the endothelium and are critical for vascular integrity. During injury endothelial cell activation causes vascular remodeling, tight junction (TJ) disruption and increased paracellular flux that disrupt brain homeostasis and aggravate disease [1, 2, 4]. Thus, augmenting vascular health and stability could be lucrative to slow disease progression and/or improve recovery. Till now however we have only limited understanding of temporal-spatial BBB alterations and mechanistic regulatory processes that occur during brain injury – obtaining better insight is an essential requirement for developing new interventions and treatment strategies.

Constant failure of acute neuroprotective strategies in stroke clinical trials emphasizes that the focus of therapeutic targets must be shifted to non-neuronal cells. Stroke heavily impacts BBB function. Stroke-induced alterations in blood flow and barrier permeability rapidly activate vascular cascades to aid re-establishment of blood flow to infarcted regions and promote recovery [5, 6]. Although it has been considered that increasing post stroke angiogenesis could accelerate recovery, implementing such a strategy in a controlled manner is very complex and unpredictable [7]. Additionally, angiogenesis, by definition, increases vascular permeability [8]—meaning taking this route might only worsen BBB compromise. Looking from the other side it is feasible that angiogenic blockade, i.e., preservation of vascular homeostasis, could be beneficial. Indeed a number of studies are now emerging to support this notion [9, 10]. As incomplete BBB recovery post stroke likely increases the risk for subsequent events and cognitive decline [5, 11], the idea that preventing BBB disturbance and endothelial cell activation will facilitate stroke recovery is increasingly reinforced.

Hypoxia-inducible factor-1 (HIF-1) regulates multiple target genes during injury conditions and its stabilization in the vascular compartments post injury dramatically alters vessel function and brain homeostasis. Many in vitro studies, including our own, convincingly show that activated HIF-1 signaling rapidly disturbs endothelial function, increases BBB permeability and modifies BBB cell-cell interactions [12–15]. In vivo data also suggests activation of HIF-1 signaling during stroke has undesirable vascular effects [16–18]. Notably, HIF-1 is induced in all cell types during stress conditions, albeit to different levels depending on cell tolerance to injury. Due to its regulation of multiple target genes during injury conditions, it is likely that HIF-1 stabilization will have divergent effects in the different BBB cellular compartments during stroke.

Pericytes are located at capillaries, pre-capillary arterioles and post-capillary venules along the vascular tree of the circulatory system, however CNS microvessels have the highest pericyte-to-endothelial cell ratio [19, 20]. Sharing a basement membrane with the endothelium means pericytes have a profound ability to regulate the vascular compartment. Indeed, they are involved in BBB formation and maintenance, vessel maturation, regulation of blood flow and immune cell trafficking as well as being a source of pluripotent stem cells (as reviewed in [2, 21, 22]). Over the last decade, interest in pericytes has increased as it is recognized that better understanding of their physiological and pathological functions could provide significant insight into vascular regulation and development of new therapeutic strategies.

Negative actions of HIF-1 on BBB stability versus its positive effects on injury adaptation in other cells, have led us to investigate how this important transcription factor alters perivascular cell responses and barrier regulation during different injury scenarios. Herein we tested whether pericyte-mediated HIF-1 signaling directly impacts barrier stability and outcome following ischemic stroke using an inducible mouse model. We show that pericytic HIF-1 deficiency reduces infarction, increases neuronal survival and suppresses barrier permeability at 72 h reperfusion. The loss of HIF-1 reduced ischemia-induced pericyte death and preserved their coverage of the CNS microvasculature translating into a more stable barrier. Importantly, this culminated in reduced neurological deficits indicating that pericyte HIF-1 stabilization *per se* compromises outcome after injury.

## Methods

### Animal experiments

 All experiments were carried out in accordance with the ‘European Convention for the Protection of Vertebrate Animals used for Experimental and other Scientific Purposes’ as well as institutional and local governmental guidelines approved by the Cantonal Veterinary Department, Zurich, Switzerland. Mice were maintained at the Nagerzentrum Tierspital, University of Zurich and housed on a 12 h light/dark cycle with access to water ad libitum and a standard laboratory diet. Animals were randomly assigned to groups by the study director who was blinded to all biometric data. Blinding was also conducted for experimenters administering treatments, assessing outcome, animal facilities staff and others who interacted with the animals.

### Generation of tamoxifen inducible pericyte-targeted HIF-1α loss of function mice (SMMHC-CreER^T2^; HIF-1α ^flox/flox^)

To obtain conditional mutant mice with pericyte-targeted HIF-1 loss of function, homozygous HIF-1α ^flox/flox^ mice with HIF-1α exon 2 flanked by loxP sites (generously provided by Randall S. Johnson [23]) were crossed with mice expressing tamoxifen-inducible Cre-recombinase under the control of the SMMHC promoter (SMMHC-CreER^T2^, generously provided by Stefan Offermanns [24]). Notably, exon 2 of the HIF-1α subunit is required for proper dimerization with HIF-1β and thus its deletion prevents formation of the functional HIF-1 complex and consequently blocks its transcriptional activity. Both lines have a C57BL/6J background and were genotyped using the primers (Microsynth, Switzerland) shown in Table S1.

Due to SMMHC-CreER^T2^ promoter being located on the Y chromosome [24], excision of the floxed HIF-1α allele was induced in adult 8–12 week-old male mice (25–30 g) by intraperitoneal injection of tamoxifen (Sigma-Aldrich, USA) dissolved in sunflower oil/ethanol mixture (50 mg/kg body weight) for 5 consecutive days. Control animals were injected with carrier only. To control for any side effects of Cre manipulation and tamoxifen treatment, SMMHC-CreER^T2^ mice were used as additional controls. On day 10–15, mice were subjected to transient middle cerebral artery occlusion (tMCAo) or sham surgery.

### tMCAo model and cerebral blood flow (CBF) monitoring

Animals were initially anesthetized with a mixture of 2 % isoflurane (Abbott, Cham, Switzerland) in an oxygen/air mixture (2:1). Subsequently, the isoflurane concentration was reduced to 1.0–1.5 % and core body temperature was maintained at 37 °C throughout surgery using a feedback-controlled heating device. To induce focal cerebral ischemia, tMCAo was performed using the intraluminal filament occlusion [25]. Briefly, the bifurcation of the left common carotid artery was exposed and a permanent ligature was made on the left external carotid artery (ECA). A 7-0-silicon monofilament (Doccol Corporation, USA) was advanced from the ECA into the internal carotid artery until it occluded the middle cerebral artery (MCA). After 45 min occlusion, the filament was removed to enable reperfusion. Laser doppler flowmetry (Moor Instruments, UK) was used to continuously measure cortical CBF in the MCA territory to ensure successful occlusion and reperfusion. After surgical procedures, animals were injected with 0.5 ml saline and monitored for 2 h. For the postoperative care, animals were subcutaneously injected with 0.5 ml 0.25 % glucose twice daily throughout reperfusion period. Animals that did not show a CBF reduction of at least 75 % from baseline level were excluded from further experimentation. For the sham groups, the procedure was performed as above but the filament was not inserted.

### Animal sacrifice and brain tissue processing

After 3 days reperfusion, individual mice were euthanized by CO_2_ inhalation and blood collected via heart puncture. The brain was removed immediately and processed accordingly. For immunostaining, the tissues were immersed in − 42 °C isopentane for 2 min then stored at − 80 °C until sectioned with a cryostat (20 μm) at eight preselected coronal levels (+ 1.98 to − 3.80 mm respect to Bregma). For molecular studies, the ischemic core, peri-infarct, and subcortex regions were isolated from the ipsilateral and contralateral hemispheres and snap frozen in liquid nitrogen until use.

### Animal preparation for magnetic resonance imaging (MRI)

Animals were anesthetized using an initial dose of 3 % isoflurane in an oxygen/air mixture (2:1), then maintained at 1.2–1.5 % isoflurane. Mice were then intubated with a polyethylene tube (inner diameter/outer diameter: 0.58/0.96 mm Portex®, Smith Medical International Ltd., UK) and actively ventilated at a rate of 90 breaths per minute (bpm) and a tidal volume of approximately 0.3 ml/breath using a small animal ventilator (MRI-1, CWE Inc., USA). Prior to MRI scanning, mice were placed in the prone position. Body temperature (36.5 ± 0.5 °C) was maintained with a water-heated support and monitored with a rectal temperature probe.

### MRI protocol

MRI scans were performed at 3 days post-stroke. Images were acquired on a 7 Tesla rodent scanner (Pharmascan 70/16 AS, Bruker BioSpin MRI GmbH, Germany). Data acquisition and image processing were performed with the Bruker software Paravision 6.0. Anatomical reference data acquired in coronal and sagittal orientations served for accurate positioning of the animal’s head using a multi-slice rapid acquisition with relaxation enhancement (RARE) spin-echo sequence [26] ranging from 3.16 mm to − 4.84 mm relative to Bregma. To quantitatively assess brain infarction, T2-weighted images were collected using a multislice Turbo-RARE sequence with the following parameters: repetition time = 2200 ms; echo time = 60 ms; RARE factor = 8; resolution = 78 × 78 μm; 10 adjacent axial slices with of 0.8 mm thickness acquired interleaved; averages = 3; field of view = 20 × 20 mm; acquisition matrix: 256 × 256 points.

### Quantification of ischemic brain damage

The outlines of contralateral hemisphere, ipsilateral hemisphere and lesion areas were delineated manually using NIH ImageJ (National Institutes of Health, USA). The total hemispheric volume of both hemispheres in lesions and regions of interest (ROIs) was obtained by integration of areas measured in ten coronal T2-weighted images using Prism-GraphPad 7.0 (GraphPad Software, USA). To correct for brain swelling, infarct volume (1) and brain edema (2) were calculated and expressed as a percentage of the contralateral hemisphere volume as below;

### Immunofluorescence staining

After 10 min 4 % paraformaldehyde fixation and washing with TBST (0.1 % Tween 20 in TBS), brain sections were blocked with 5 % normal goat serum, 0.3 % Triton X-100 in TBS for 2 h at room temperature, then incubated with primary antibodies: Cre-recombinase (1:100, Millipore, USA), PDGFR-β (1:500, Santa Cruz, USA), NG-2 (1:500, Millipore, USA), NeuN (1:1000, Millipore, USA), GFAP (1:1000, Sigma-Aldrich, USA), CD31 (1:1000, BD Bioscience, USA), Claudin-5 (1:250, Invitrogen, USA) and ZO-1 (1:100, Invitrogen, USA) at 4 °C overnight followed by fluorophore conjugated-secondary antibodies (1:500, Thermo scientific, USA) for 60 min. Cell nuclei were counterstained with DAPI.

### TdT-mediated dUTP nick-end labelling (TUNEL) assay

TUNEL staining kit was used to detect fragmented nuclear DNA during apoptosis according to the manufacturer instructions (Roche, Switzerland). Following permeabilization with 0.1 % Triton-X 100 in 0.1 % sodium citrate, fixed sections were incubated with TUNEL reaction mix at 37 °C for 60 min. Washed slides were then immunostained and counterstained with DAPI.

### Fluoro-Jade C staining (FJC)

To visualize degenerative neurons, brain sections were subjected to FJC staining according to kit instructions (Millipore, USA). Briefly, the slides were fixed with 4 % paraformaldehyde for 10 min then immersed in 1 % NaOH in 80 % ethanol for 5 min. After rinsing for 2 min each in 70 % ethanol and ddH_2_O, slides were incubated in 0.06 % potassium permanganate solution for 10 min, rinsed in ddH_2_O, then transferred to the staining solution for 10 min. Post washing, slides were air-dried at 50 °C for 30 min then cleared in xylene and mounted with Entellan New (Sigma-Aldrich, USA).

### IgG extravasation staining

For measurement of endogenous IgG leakage, two brain sections at level − 0.38 and − 1.28 mm relative to Bregma were used. Sections were blocked in 10 % normal goat serum for 2 h at room temperature followed by 1 h incubation with the biotinylated secondary anti-mouse IgG antibody (1:500, Jackson ImmunoResearch, USA) and 1 h with streptavidin/horseradish peroxidase antibody (1:500 Jackson ImmunoResearch, USA). After exposure to diaminobenzidine (DAB) solution (0.05 % DAB in 0.005 % H_2_O_2_) and mounting, extravasation was calculated as fold change increase of IgG intensity in the ipsilateral cortex/subcortex compared to the corresponding contralateral area.

### Image acquisition and analysis

Images were acquired with an epifluorescence microscope (Carl Zeiss, Germany) at 20x magnification spanning three ROIs including ischemic core, peri-infarct and subcortex. Three images were taken from selected regions. Two consecutive sections for each animal were used. The average of three images was calculated for each selected region. For TUNEL, FJC and NeuN analysis, numbers of positive cells were counted on 20x microscopy pictures from selected ROIs using NIH ImageJ with threshold processing and particle analysis. All data was expressed as cell numbers or areas per mm^2^.

To obtain blood vessel parameters, the binary images generated by threshold processing were analysed with ImageJ plugin Skeletonize (2D/3D) and Analyze Skeleton (2D/3D). Pericyte coverage was calculated as the area of overlap of NG-2 positive areas of pericytes versus CD31-positive areas of endothelial cells. To determine pericyte death, TUNEL and NG-2 double-labelled cells were counted manually and the percentage calculated as double positive cells versus total TUNEL-positive cells.

### Evans blue leakage assay

At 3 days post-surgery, prior to sacrifice animals were intravenously injected with 1 % Evans blue dye (2 µg/g body weight) via the femoral vein. After 45 min circulation, animals were anesthetized and transcardially perfused with ice cold PBS. Ipsilateral and contralateral hemispheres were collected and dye retained in the tissue extracted with formamide (5 µl/mg tissue weight) for 72 h. Absorbance was measured at 620 nm and leakage calculated via a standard curve and expressed as fold change of the contralateral hemisphere baseline.

### Cytosolic and nuclear fraction and Cre-recombinase localization

 Western blotting was performed with fractioned brain proteins. Briefly, half brains were homogenized with a dounce homogenizer in ice cold homogenization buffer (0.27 M sucrose, 2mM EDTA pH8, 0.06 M KCl, 15mM NaCl, 15mM HEPES) supplemented with 1mM phenylmethansulfonyl fluoride (PMSF, Sigma-Aldrich, USA), 1mM sodium orthovanadate (NaV, Sigma-Aldrich, USA), and proteinase inhibitor cocktails (Calbiochem, Germany). The cell suspension was layered onto a sucrose cushion (30 % w/v sucrose, 2mM EDTA pH8, 0.06 M KCl, 15mM NaCl, 15mM HEPES) and centrifuged at 1500x*g* for 10 min at 4 °C. The cytosolic fraction was collected and the nuclear pellet resuspended in extraction buffer (20mM HEPES, 400mM NaCl, 1mM EDTA pH8) supplemented with 1mM PMSF, 1mM NaV and a proteinase inhibitor cocktail. After 15 min incubation on ice, the nuclear fraction was obtained by centrifugation (15,000x*g* for 5 min) at 4 °C. Proteins (30 µg) were separated by SDS-PAGE then transferred to 0.45 μm nitrocellulose membranes (Sigma-Aldrich, USA). After blocking in 5 % non-fat dry milk in TBS, membranes were incubated with primary antibodies against Cre-recombinase and β-actin (MAB3120 and A5441 respectively, Sigma-Aldrich, USA). Following incubation in horse radish peroxidase (HRP)-conjugated secondary antibodies for 1 h at RT, bands were detected using a luminescent image analyzer (Fujifilm, LAS-3000, Japan).

### Protein extraction and immunoblotting

Ischemic core and peri-infarct regions were dissected from ipsilateral hemispheres then suspended in RIPA buffer (50mM Tris HCl, PH 7.4,150mM NaCl, 1 % NP-40, 0.5 % Sodium deoxylcholate,0.1 % SDS, 1mM EDTA,10mM NaF) with protease inhibitors (Thermo scientific, USA) and homogenized with a dounce homogenizer. 30–40 µg protein was separated on denaturing SDS-PAGE and transferred to a nitrocellulose membrane. Membranes were blocked in 5 % non-fat dried milk or 5 % BSA for 60 min, then incubated overnight at 4 °C in primary antibodies against β-actin (1:5000, Sigma-Aldrich), Occludin (1:500, Invitrogen, USA), Claudin-5 (1:250, Abcam, UK), ZO-1 (1:250, Invitrogen, USA), β-catenin (1:1000, BD Bioscience, USA) and VE-Cadherin (1:1000, Santa Cruz, USA). Following incubation with a HRP-conjugated secondary antibody, band detection was performed using a luminescent image analyzer LAS-3000 (Fujifilm, Japan) then quantified with NIH ImageJ software using β-actin as a loading control. Fold change was calculated by normalizing to Sham-Ctrl animals.

### Neurological scoring

Clark’s deficit scoring [27] was performed 1 day before tMCAo/sham surgery and on day 1 and 3 post-surgery. The score is comprised of two grading scales: (i) A general deficit scale evaluating hair, eyes, ears, posture, spontaneous activity and epileptic behavior and (ii) A focal deficit scale evaluating body symmetry, gait, climbing on a surface held at 45º, circling behavior, front limb symmetry, compulsory circling and whisker response. Scores for each scale range from 0 to 28 with the sum of each category presented. Animals with a score > 21 on the focal deficit scale were excluded from the study.

### Neurobehavioural testing

Three different tests were performed 1 day prior to tMCAo/sham surgery then on day 3 post-surgery. The ladder rung task, adapted from a rat model [28], is designed to assess post stroke sensorimotor function by measuring foot placement, stepping and inter-limb coordination. The apparatus is composed of two Plexiglas walls (69.5 × 15 cm, spaced 5 cm apart). Each wall has 121 holes of 0.2 cm in diameter, spaced 0.5 cm apart and located 1 cm from the bottom of the wall filled with metal bars (8 cm in length, diameter 0.1 cm). The apparatus is placed on top of two standard mouse cages. Animals were tested with the rungs in an irregular pattern with a varied distance from 0.5 to 2.5 cm. Each test included two crossings with performance video-recorded and analysed frame-by-frame with each limb scored for placement with every step according to a predetermined scale. The corner test identifies sensorimotor and postural asymmetries and is considered an objective assessment of long-term outcome after stroke [29]. To start the trial, the mouse is placed halfway between two angled (30º) boards facing the corner. On entering deep into the corner both sides of the vibrissae are stimulated causing the mouse to rear forward and upward and turn back to face the open end. Unimpaired animals turn either left or right whereas injured animals preferentially turn toward the non-impaired side. Turns in one versus the other direction were recorded from ten trials with turning movements not part of a rearing movement being discarded. The latency to move test was used to highlight altered locomotor activity post stroke [30]. The test plate is composed of two Plexiglas walls (15 × 7 cm) spaced 5 cm apart producing a corridor a mouse can walk through but not easily turn back. Animals are placed in front of the test plate entrance and the time to walk through the corridor was recorded. Each test was performed three times and the mean value calculated.

### Statistical analysis

Statistical analysis was performed with GraphPad Prism 7.0 (GraphPad Software, USA). All results were shown as mean values ± standard deviation with a minimum of 5 independent experiments. Statistical significance was determined by two-way ANOVA with Bonferroni post-hoc test when comparing sham-animals and stroke animals or unpaired student’s t-test with heteroscedasticity when comparing stroke only groups. A p value less than 0.05 was considered statistically significant.

## Results

### Generation and characterization of SMMHC-CreER ^T2^; HIF-1α^flox/flox^ mouse line

**Fig. 1:**
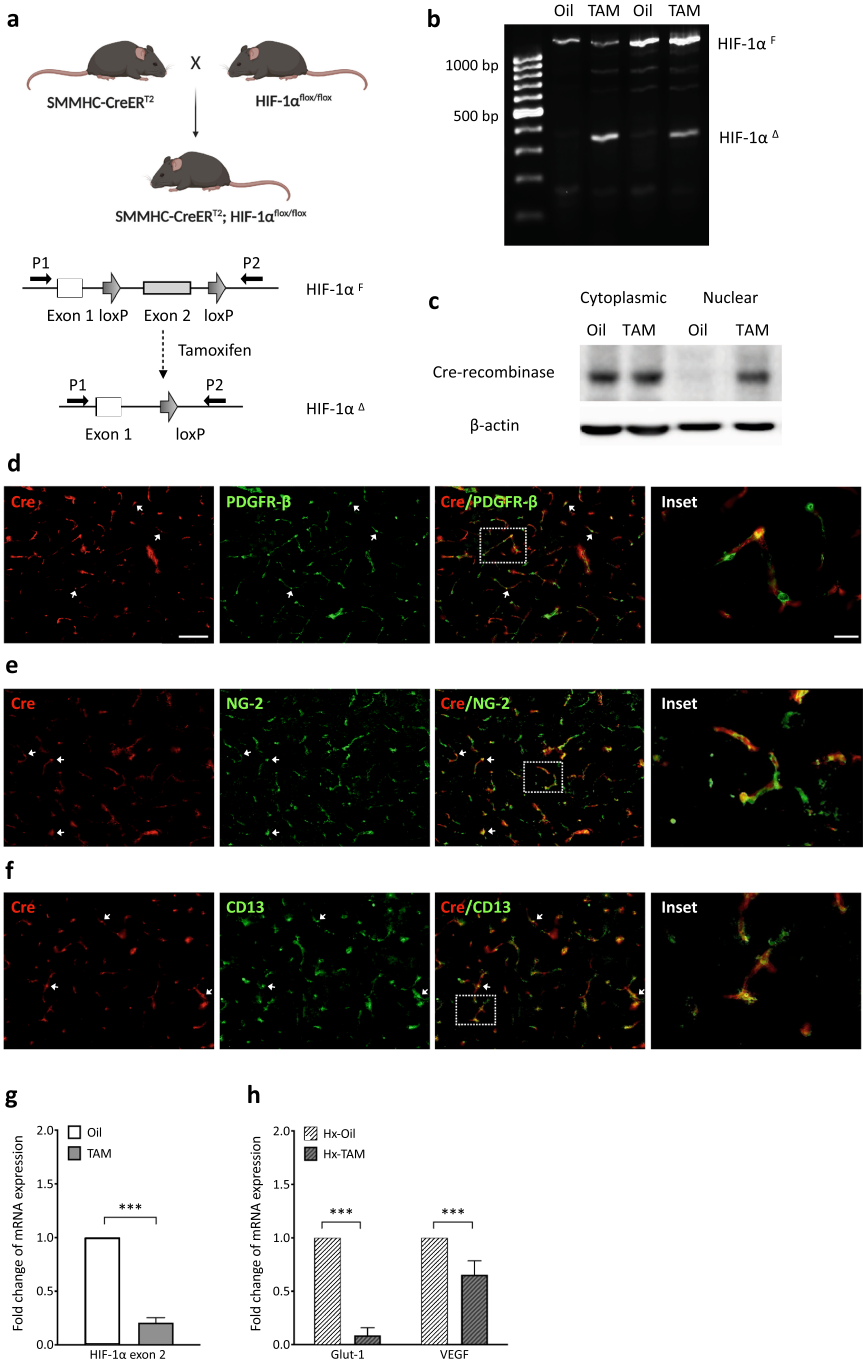
Generation and characterization of SMMHC-CreER ^T2^; HIF-1α ^flox/flox^ mouse line. **a** Schematic representation of conditional HIF-1 gene disruption. Since HIF-1α exon 2 is floxed, tamoxifen treatment induces Cre-mediated recombination and exon 2 deletion. P1: primer 1; P2: primer 2; HIF-1α^F^: floxed HIF-1α allele; HIF-1α^Δ^: HIF-1α exon 2 deleted allele. **b** PCR-based confirmation of Cre-mediated recombination in brain cortices isolated from SMMHC-CreER^T2^; HIF-1α^flox/flox^ mice after vehicle (Oil) or tamoxifen (TAM) injection with primers designated in schematic (**a).** Tamoxifen treatment generates a short 300 bp band (HIF-1α^Δ^) compared to the oil control 1.1Kb fragment (HIF-1α^F^) allele. **c** Cre-recombinase nuclear translocation is mediated by tamoxifen treatment as observed by immunoblotting of cytoplasmic and nuclear protein fractions. β-actin is the loading control. **d–f** Double-staining of brain sections with pericyte markers PDGFR-β, NG-2 or CD13 (green) and Cre-recombinase (red) confirms Cre-recombinase expression in brain pericytes. Arrowheads highlight vascular localization of PDGFR-β (**d**), NG-2 (**e**) and CD13 (**f**) positive cells. Scale bar = 100 μm. Inserts are 1.6x magnified images of boxed regions. **g** Quantitative RT-PCR of HIF-1α exon 2 expression in primary brain pericytes isolated from SMMHC-CreER^T2^; HIF-1α^flox/flox^ mice exposed either to tamoxifen (2 µM) or vehicle (Oil) for 48 h. **h** Analysis of gene expression of HIF-1 targets in primary pericytes with or without tamoxifen treatment after 48 h hypoxia (Hx, 1 % O_2_). Glut-1: Glucose transporter 1, VEGF: Vascular endothelial growth factor. Unpaired student’s *t* test *P < 0.05; **P < 0.01; ***P < 0.001. Mean ± SD. *n* = 3–4

We generated inducible pericyte-targeted HIF-1 loss of function (LoF) mice (SMMHC-CreER^T2^; HIF-1α^flox/flox^ mouse line) by crossing HIF-1α floxed mice (HIF-1α^flox/flox^) [23] with SMMHC-CreER^T2^ mice [24] as shown in Fig. 1a. After intraperitoneal tamoxifen injections followed by 5 days recombination period, Cre-mediated recombination was confirmed by PCR analysis of genomic DNA isolated from brain cortices. Full length floxed HIF-1α allele (HIF-1α^F^) was seen in oil-treated groups and tamoxifen treatment caused a truncated excision product (HIF-1α^Δ^) (Fig. 1b). In correlation, Western blotting revealed nuclear translocation of Cre-recombinase only in tamoxifen-injected groups (Fig. 1c). Mouse brain sections co-stained with Cre and pericyte markers (PDGFR-β, NG-2 and CD13) showed the recombinase is robustly expressed in areas surrounding and/or juxtaposed to the vasculature. Moreover, Cre expression was largely restricted to PDGFR-β, NG-2 and CD13 positive-pericytes (Fig. 1d–f). Functional confirmation of HIF-1 LoF was performed by qRT-PCR analysis on RNA from primary mouse brain pericytes isolated from the line after treatment with 2 µM tamoxifen or oil for 48 h. Upon tamoxifen treatment, HIF-1α exon 2 mRNA expression was markedly decreased when compared with the oil group (Fig. 1g). Moreover, hypoxic (8 % O_2_ for 48 h) exposure of primary pericytes resulted in markedly reduced expression of HIF-1 target genes Glut-1 and vascular endothelial growth factor (VEGF) post tamoxifen treatment (Fig. 1h).

### Ischemic severity is abrogated in pericyte HIF-1 LoF animals

Hypoxia/Ischemia disturbs BBB functionality [12–15]. As barrier disturbance negatively affects stroke outcome, we asked if pericytic HIF-1 signaling influences brain infarction post stroke. We subjected our SMMHC-CreER^T2^; HIF-1α^flox/flox^ mice to tMCAo or sham surgery for 45 min using the intraluminal method [25] after oil (Ctrl) or tamoxifen treatment (HIF-1 LoF). CBF was measured by Laser Doppler flowmetry in the brain cortex throughout the entire surgical procedure to ensure success. As expected, MCA occlusion led to a significant reduction of cortical CBF in both stroke groups (Stroke-Ctrl: 78.17 ± 5.74 %; Stroke-HIF-1 LoF: 76.51 ± 4.69 %; Supplementary Fig. 1) as well as in additional tamoxifen/oil treated SMMHC-CreER^T2^ controls (Stroke-Oil: 79.00 ± 5.08 %; Stroke-TAM: 80.00 ± 2.29 %; Supplementary Fig. 1). Flow returned to within 50 % of original values upon withdrawal of the inserted filament (reperfusion) and virtually recovered to baseline by the end of surgery. All animals showed the same CBF profile during the tMCAo procedure.

**Fig. 2:**
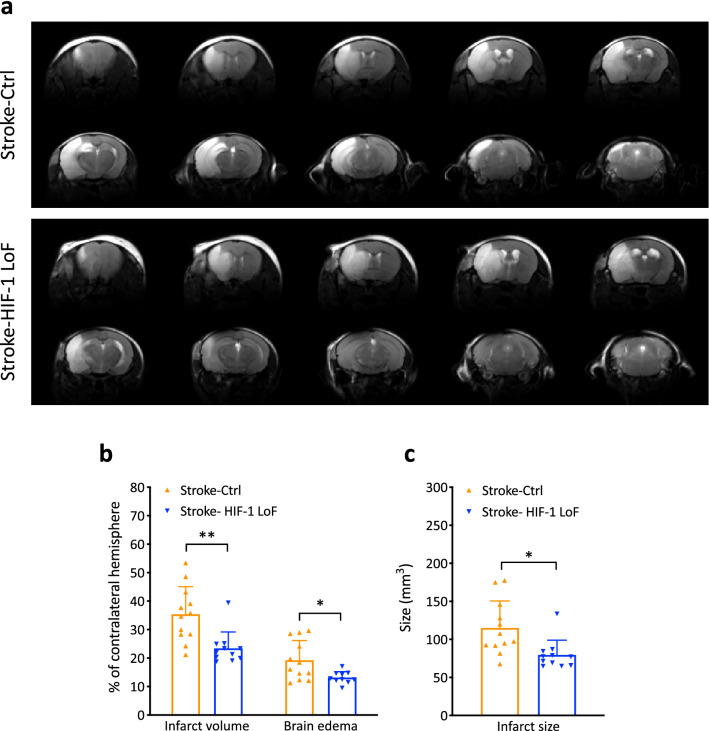
Loss of pericyte HIF-1 function reduces brain damage. **a** T2-weighted MRI scanning was used to determine brain infarction at 3 days post stroke. Representative consecutive coronal T2-weighted images of Stroke-Ctrl and Stroke-HIF-1 LoF animals, hyperintense (bright) areas indicate the lesion. **b** Histogram of infarct volume and brain edema in % of contralateral hemispheric volume **c** Total infarct size (not corrected for brain swelling) calculated via integration of under the curve analysis. Unpaired *t* test *P < 0.05; **P < 0.01; Mean ± SD. *n* = 11–13

Brain infarction, monitored via T2-weighted MRI scanning at 3 days post stroke, revealed large brain lesions throughout the ipsilateral hemisphere of the control group (visualized by hyperintense (bright) areas in Fig. 2a). Notably, lesion size looked similar in the anterior brain of Stroke-HIF-1 LoF mice whereas reduced infarct volumes were observed in the posterior regions ranging from − 0.84 mm to -4.84 mm relative to Bregma compared to control animals (Fig. 2a). Quantification showed that infarct volume, brain edema, and infarct size are significantly reduced in Stroke-HIF-1 LoF mice compared to controls (Fig. 2b, c). Thus, abrogation of pericyte HIF-1 signaling attenuates infarct severity post stroke.

### Pericyte HIF-1 LoF attenuates neuronal damage in peri-infarct regions

**Fig. 3:**
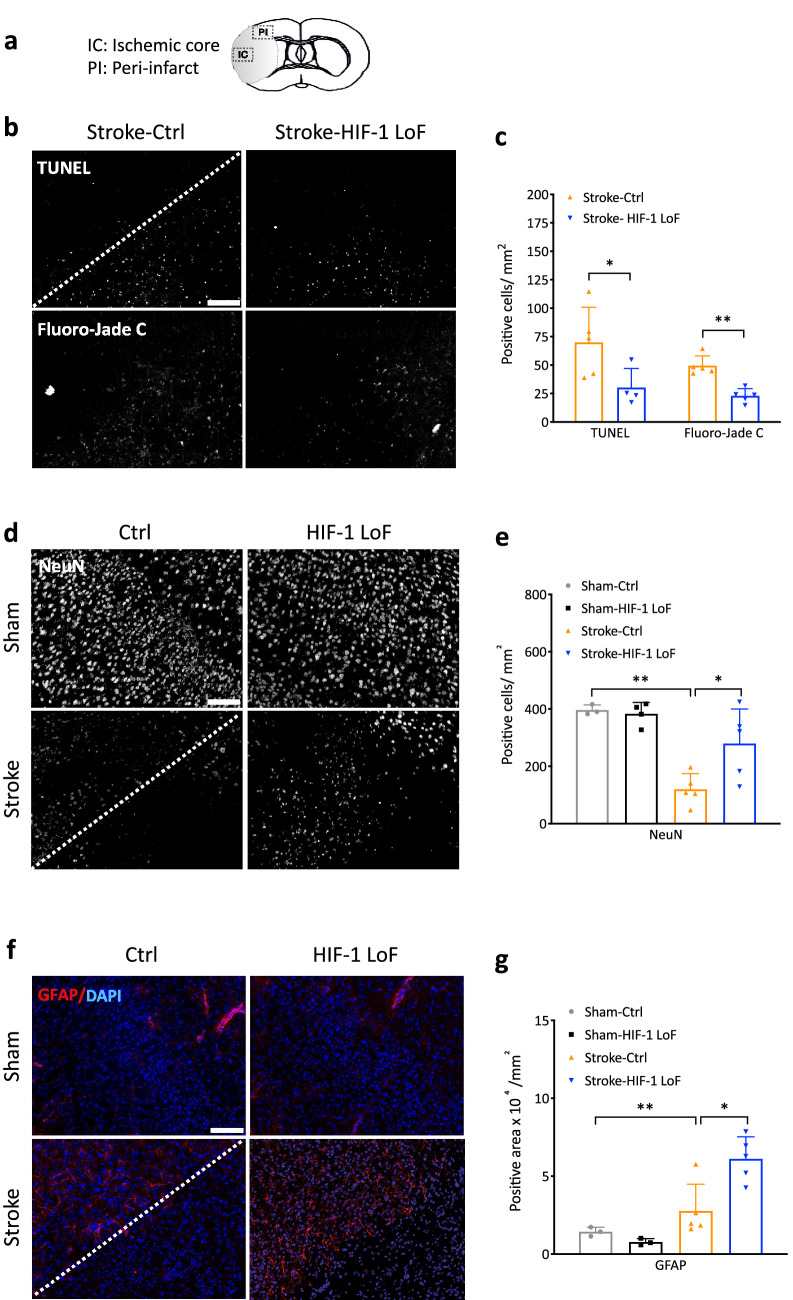
Pericyte HIF-1 LoF attenuates ischemic severity of the peri-infarct area. **a** Schematic of coronal brain section showing regions-of-interest (ROIs) for quantitative analysis. **b–c** Representative images (**b)** and quantification (**c**) of TUNEL (apoptotic cells) and Fluoro-Jade C (degenerated neurons) staining in the peri-infarct area of stroke animals at 3 days after surgery. **d, e** Representative images **(d)** and quantification **(e)** of NeuN (neuronal nuclei) staining in peri-infarct area of stroke and sham animal groups. **f**,** g** Representative images **(f)** and quantification **(g)** of GFAP (astrocytes) staining of stroke and sham animal groups. Cell nuclei are counterstained with DAPI (blue). The border between the peri-infarct area (upper left) and ischemic area (lower right) is indicated by the white dotted line. Scale bars = 100 μm. Unpaired t-test vs. Stroke-Ctrl group; *P < 0.05; **P < 0.01; Two-way ANOVA for comparison of four groups. *P < 0.05; **P < 0.01 Mean ± SD. *n* = 4–6

Stroke causes irreparable damage to most cells in the ischemic core but cells in peri-infarct regions are considered to be salvageable. We assessed whether ischemia-induced damage within these ROIs, i.e. ischemic core and peri-infarct areas (Fig. 3a), is rescued by pericyte HIF-1 LoF in correlation with reduced stroke severity. Overall cell damage was evaluated by TUNEL analysis and the extent of neuronal degeneration and survival was specifically assessed by FJC and NeuN staining, respectively. In the ischemic core, considerable apoptosis and neuronal degeneration occurred in both stroke groups to a similar degree as confirmed by quantification (Supplementary Fig. 2a&b). In peri-infarct regions, most TUNEL-positive cells were located in areas immediately adjacent to the ischemic areas (Fig. 3b) with significantly less apoptotic cells detected in Stroke-HIF-1 LoF animals compared to controls. (Fig. 3c). Notably, there was also reduced neuronal degeneration in HIF-1 LoF animals compared to controls (Fig. 3b&c). In correlation, NeuN staining showed abrogating HIF-1 improved neuronal survival (Fig. 3d&e).

Astrocyte activation in peri-infarct regions is a hallmark during the subacute phase of stroke and beneficial to stroke progression and outcome [31]. Hypertrophic astrocytic processes and stellate morphology were highly evident in peri-infarct regions of both stroke mice by GFAP immunohistochemistry (Fig. 3f). However, quantification revealed more wide-spread GFAP-positive areas in Stroke-HIF-1 LoF animals compared to HIF-Ctrl mice (Fig. 3g). Although post stroke inflammatory responses were also monitored via CD11b and Iba1 staining, no difference in the number of activated microglia or infiltrating macrophages were noted between the stroke groups (data not shown).

### Pericyte HIF-1 stabilization mediates stroke-induced barrier permeability

**Fig. 4:**
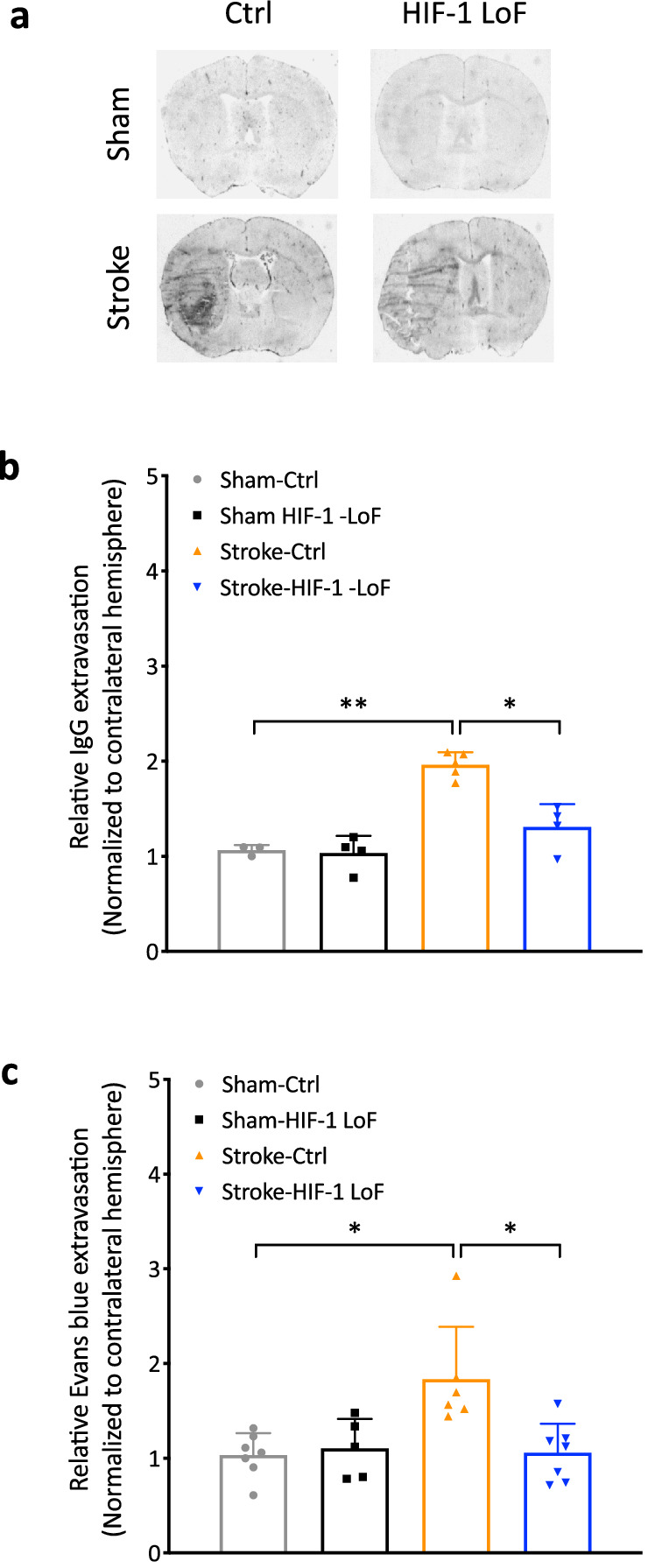
Decreased stroke-induced barrier permeability upon loss of HIF-1 function. **a**,** b** Representative images **(a)** and quantification **(b)** of IgG extravasation in stroke and sham groups. **c** Quantification of Evans blue dye extravasation into brain tissue. Relative change of barrier leakage was obtained by normalizing to values of the contralateral hemisphere. Two-way ANOVA *P < 0.05; **P < 0.01; Mean ± SD. *n* = 4–6

We next asked if attenuation of stroke severity and neurological damage observed in the LoF mice could be a consequence of sustained barrier integrity during injury. To evaluate barrier permeability, we first measured IgG extravasation by immunostaining at 3 days post stroke. As expected, and in line with published data, stroke-induced IgG extravasation was observed in ipsilateral but not contralateral hemispheres of stroke mice (Fig. 4a). Quantification of the staining further showed Stroke-HIF-1 LoF mice have significantly less IgG leakage compared to Stroke-Ctrl animals (Fig. 4b). These findings were corroborated by functional assessment of vascular leakage using Evans blue as reduced dye extravasation was also measured in Stroke-HIF-1 LoF mice compared to Stroke-Ctrl animals. Notably, Stroke-HIF-1 LoF groups exhibited extravasation levels that were similar to sham-operated mice (Fig. 4c) indicating a strong impact on barrier functionality. Clearly, improved barrier integrity in pericytic HIF-1 LoF mice correlates with reduced injury severity.

### Pericyte HIF-1 improves tight junction arrangement in peri-infarct regions

**Fig. 5:**
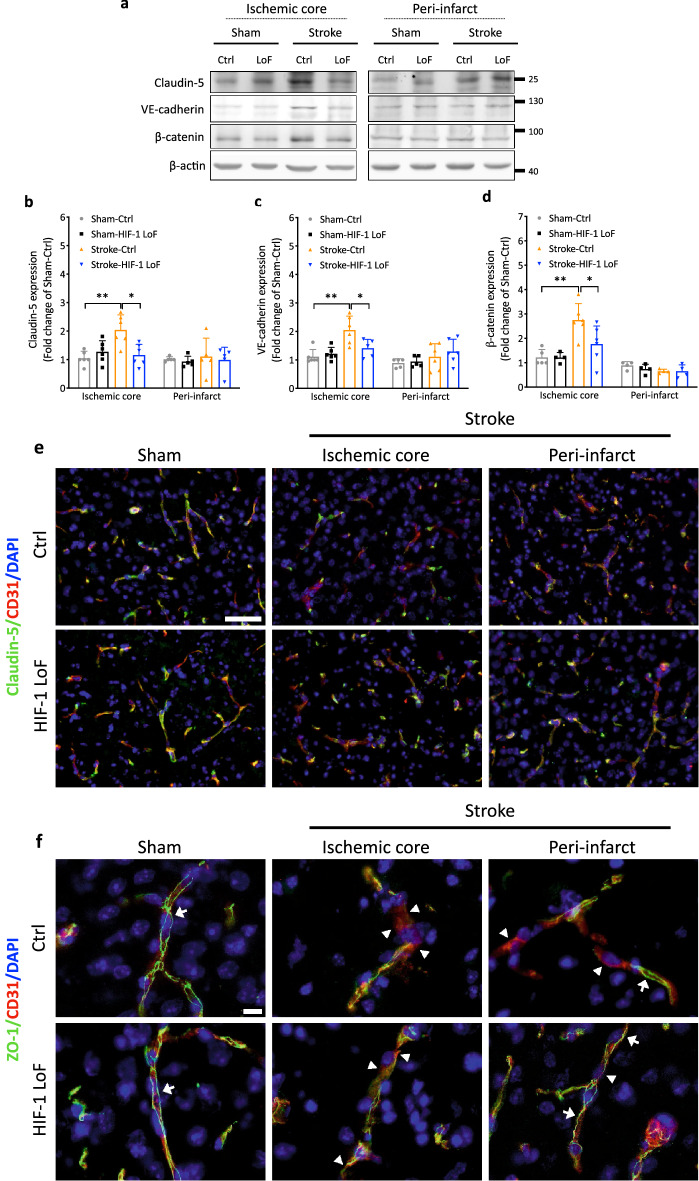
Pericyte HIF-1 LoF sustains tight junction organization in peri-infarct regions. **a–d** Representative immunoblots (**a**) and quantification of Claudin-5 (**b**), VE-cadherin (**c**) and β-catenin (**d**) protein levels in the ischemic core and peri-infarct areas of sham and stroke animals 3 days post surgery. β-actin is the loading control. Two-way ANOVA; *P < 0.05; **P < 0.01; Mean ± SD. *n* = 3–6. **e-f** Representative images of ischemic core and peri-infarct areas from brain sections of sham and stroke animals co-immunostained for CD31 and the junctional proteins Claudin-5 (**e**) and ZO-1 (**f**). Nuclei are counterstained with DAPI. Scale bar = 100 μm. Arrows indicate intact continuous TJ staining whereas arrowheads highlight disrupted regions at vessel walls

Tight and adherens junction complexes are guardians of the barrier function per se [2, 4]. Since loss of pericyte HIF-1 prevented stroke-induced barrier permeability, we evaluated expression of key junctional proteins post surgery by Western blotting. In the ischemic core, a significant induction of Claudin-5 expression was observed in Stroke-Ctrl mice versus Sham-Ctrl and Stroke-HIF-1 LoF mice (Fig. 5a, b). Levels of VE-cadherin, an adherens junction (AJ) component that regulates Claudin-5 expression via β-catenin signaling [32], were also induced in correlation with increased β-catenin expression (Fig. 5c, d) in Stroke-Ctrl mice. Notably, TJ and AJ protein levels in Stroke-HIF-1 LoF mice were comparable to those of sham animals. Overall, there were no significant differences in infarct ZO-1 or Occludin levels in any of the groups although a trend to decrease was induced by ischemia. (Supplementary Fig. 3a–c). Surprisingly, in the peri-infarct, stroke did not dramatically impact expression levels of any of these TJ or AJ proteins (Fig. 5a–d and Supplementary Fig. 3a–c), an unprecedented observation considering the large differences in permeability. Thus to understand why Stroke-HIF-1 LoF mice had better BBB functionality we performed immunohistochemistry to visualize Claudin-5 (Fig. 5e) and ZO-1 (Fig. 5f) organization at the vessel walls. The low power Claudin-5 images provide an excellent overview of regional localization and vessel structure whereas the high power ZO-1 images highlight TJ organization *per se*. In the core area, ischemia induced a clear loss of TJ expression at the vessel walls and considerably disrupted overall vessel structure to a similar extent in both Ctrl and LoF mice. However, in peri-infarct regions a noticeable difference between the animals was noted. Whereas Ctrl mice still presented largely disturbed vessels and TJ delocalization, the LoF mice had discernably better TJ organization with vessel structures more akin to that of sham operated animals (Fig. 5e, f). These findings correlate well with sustained barrier functionality in Stroke-HIF-1 LoF mice and show that HIF-1 LoF improves TJ organization in peri-infarct microvessels without modulating expression levels.

### Vascular alterations are abrogated by pericyte HIF-1 deletion

**Fig. 6:**
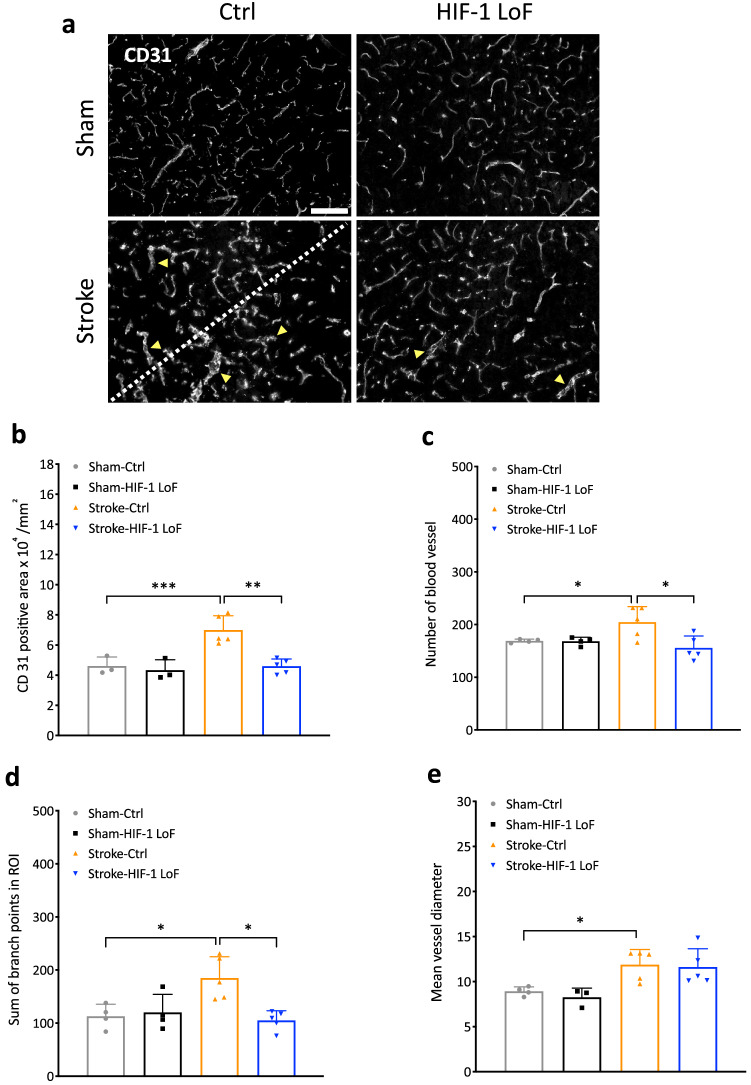
Pericytic HIF-1 deficiency abrogates post stroke vascular remodeling. **a** Representative images of CD31 staining in the peri-infarct of all animal groups at 3 days reperfusion. Scale bar = 100 μm. Arrowheads mark clearly dilated vessels. The border between peri-infarct areas and ischemic regions is indicated by the white dotted line. **b–e** Quantification of CD31 positive area (**b**), number of blood vessels (**c**), total branch points (**d**) and mean vessel diameter (**e**). Two-way ANOVA *P < 0.05; **P < 0.01; ***P < 0.001; Mean ± SD. *n* = 4–6

As vascular alterations such as vasodilation or angiogenesis occur during stressful conditions such as hypoxia or ischemia [2], the reduced barrier permeability observed in HIF-1 LoF mice might be attributed to stabilization of the vasculature. To address this, we used CD31 staining to assess vascular morphological changes. In the ischemic core, fewer CD31-positive blood vessels (Supplementary Fig. 4a) and reduced CD31-positive area compared to sham-operated animals was noted (Supplementary Fig. 4b). Significant increases in vessel diameter (vasodilation) in both stroke groups were also apparent (Supplementary Fig. 4c). In peri-infarct regions, both vasodilation and disordered vessel organization were clearly visible in Stroke-Ctrl mice (highlighted by arrowheads) whereas the vessel structure of Stroke-HIF-1 LoF mice was much more comparable to sham-operated mice (Fig. 6a). Quantification analysis showed significant increases in CD31-positive area, vessel number and total vessel branch points in Stroke-Ctrl mice compared to sham controls indicating the occurrence of stroke-induced angiogenesis. Stroke-HIF-1 LoF mice on the other hand did not display any increment in these parameters (Fig. 6b–d). Collectively, the data suggests Stroke-HIF-1 LoF mice have a more stable peri-infarct vasculature post surgery. These effects seem not to be mediated by the classic HIF-1 target VEGF as an ELISA assay showed that despite being clearly induced by stroke, there was no difference in infarct core, peri-infarct or serum VEGF levels between Ctrl or LoF mice (data not shown).

### Loss of HIF-1 signaling maintains vessel pericyte coverage

**Fig. 7:**
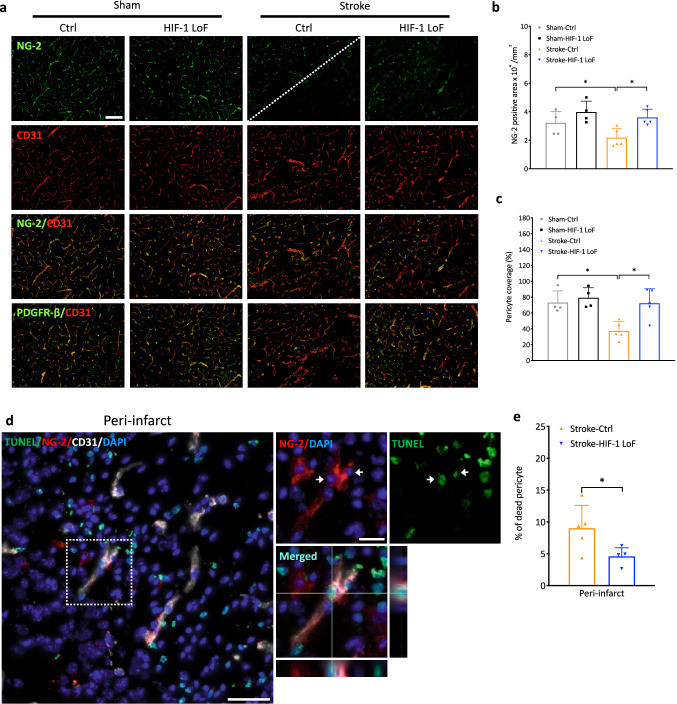
Loss of HIF-1 signaling prevents pericyte death and improves coverage. **a** Representative images of NG-2 or PDGFR-β (green; pericytes) and CD31 (red; endothelial cells) staining in peri-infarct areas at 3 days reperfusion. The border between the peri-infarct area and ischemic core is indicated by a white dotted line. **b-c** Histograms of NG-2 positive areas (**b**) and pericyte coverage (**c**) i.e. the area of overlap of NG-2-positive pericytes and CD31-positive endothelial cells. Scale bar = 100 μm. **d** Representative images showing pericyte death in the peri-infarct region of Stroke Ctrl mice. Sections are stained with TUNEL (green), NG-2 (red), CD31 (white) and counterstained with DAPI (blue). The images on the right are 1.6X magnifications of the boxed region with TUNEL/NG-2 double positive cells within a vessel highlighted with arrowheads. Merged orthogonal views of horizontal and vertical Z-stack images confirm localization of TUNEL to the nucleus of an NG-2 positive pericyte. Scale bars = 50 μm. **f** Quantification of % pericyte death in peri-infarct areas of Stroke mice groups. Unpaired t-test compared to Stroke-Ctrl *P < 0.05; Two-way ANOVA for comparison between four groups. *P < 0.05 Mean ± SD. *n* = 4–6

The reduction or detachment of pericytes from blood vessels after stroke correlates with increased BBB permeability [33, 34]. To evaluate if HIF-1 impacts pericyte localization we used PDGFR-β and NG-2 co-immunostaining to visualize pericyte coverage and then calculated the area of overlap with CD31-positive endothelial cells. In addition, NG-2-positive areas were measured to roughly compare pericyte numbers. In the sham-operated animals, PDGFR-β and NG-2 positive pericytes were closely associated with and wrapped around blood vessels in regions corresponding to the ischemic core (Supplementary Fig. 5a) and peri-infarct area (Fig. 7a). Pericyte coverage of the microvasculature was approximately 70–80 % in both regions (Fig. 7c and Supplementary Fig. 5c respectively), an observation consistent with other studies [33, 35]. In the ischemic core, less pericytes and endothelial cells were detected in stroke animals compared to sham groups, and numerous blood vessels were completely unsheathed or only slightly covered by pericytes (Supplementary Fig. 5a). Reduced NG-2 positive areas in both stroke groups compared to sham controls (Supplementary Fig. 5b) suggested considerable pericyte loss in the ischemic core. In line, pericyte coverage was significantly decreased in Stroke-Ctrl versus Sham-Ctrl animals. Stroke-HIF-1 LoF mice showed a trend to higher pericyte coverage that was not statistically different compared to Stroke-Ctrl (Supplementary Fig. 5c). In peri-infarct regions, contact between some CD31-positive blood vessels and PDGFR-β or NG-2-positive pericytes was seen beyond the ischemic border (indicated by a dotted line) of Stroke-Ctrl mice (Fig. 7a). These pericyte-covered vessels had considerably better morphology compared to the non-covered ones adjacent to infarct core areas. In Stroke-HIF-1 LoF animals, the majority of blood vessels, indeed even those at the ischemic border, were regularly covered by pericytes (Fig. 7a). The increased degree of pericyte coverage compared to control animals was clearly apparent after quantification as both NG-2-positive area (Fig. 7b) and pericyte coverage (Fig. 7c) were consistently maintained in Stroke-HIF-1 LoF mice. To understand if this observation was due to reduced pericyte death in Stroke-HIF-1 LoF mice, brain sections were co-stained with NG-2 or PDGFR-β, CD31 and TUNEL (Fig. 7d and Supplementary Fig. 5d). The first panels show an overview of co-stained vessels in the peri-infarct brain region. The boxed vessel is magnified in subsequent images and pericytes that are also TUNEL positive are highlighted with arrows (Fig. 7d and Supplementary Fig. 5d). Reconstruction of merged vertical and horizontal z-stack orthogonal images of a single cell further emphasizes co-localization. Notably, in HIF-1 LoF mice TUNEL positive pericytes were only sparsely identified in peri-infarct regions as confirmed by quantification of significantly less pericyte death (Fig. 7e). Thus, blockade of HIF-1 signaling prevents pericyte loss post stroke.

### HIF-1 LoF mice have reduced neurological deficits

**Fig. 8:**
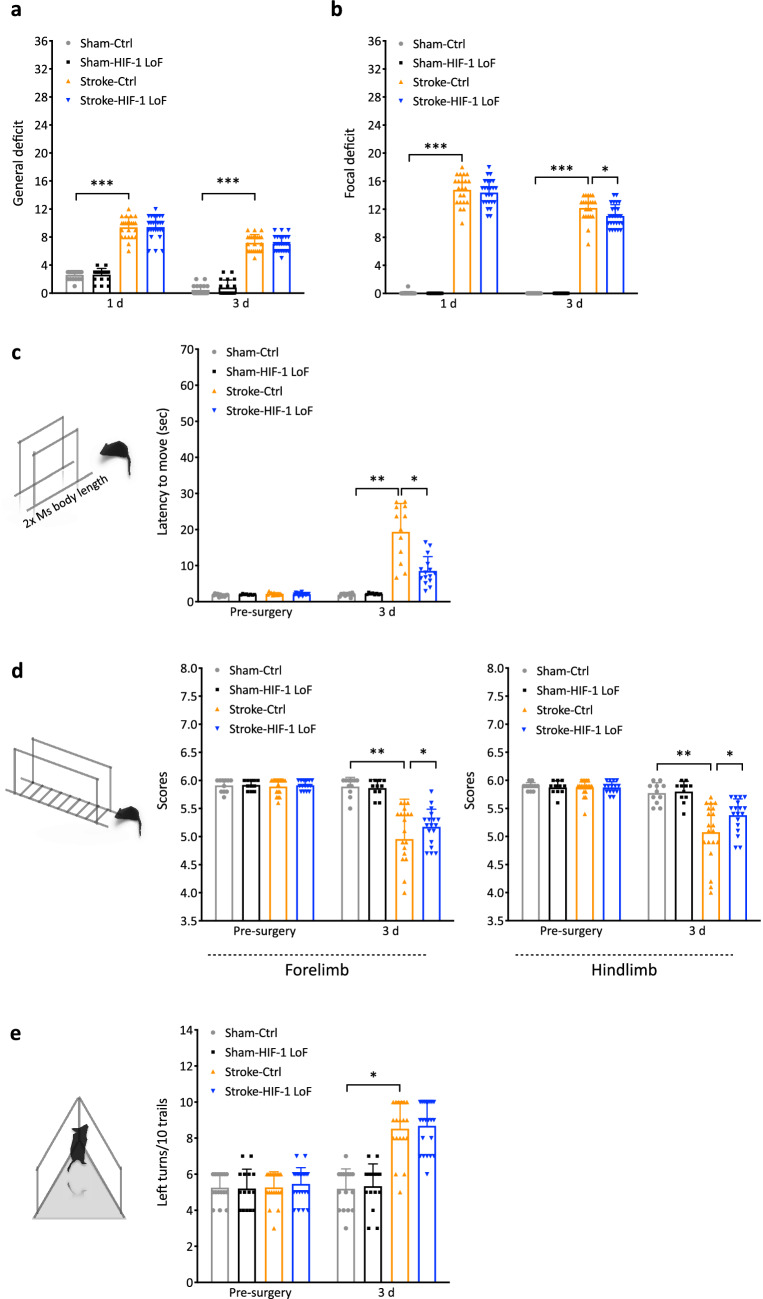
HIF-1 LoF mice show reduced neurological deficits post surgery. Graphs of Clark’s score general (**a**) and focal (**b**) deficits at 1 day and 3 days post tMCAo or sham surgery. Neurobehavioural assessment of sensorimotor coordination using latency to move test (**c**), ladder rung test (**d**) and corner test (**e**) 1 day prior to surgery and after 3 days reperfusion. The schematics show the testing platform for each behavioural assessment. Ms: mouse. Two-way ANOVA. *P < 0.05, *P < 0.01 Mean ± SD. *n* = 12–14 for sham-operated animals; *n* = 16–18 for stroke groups

Less ischemic damage and a better-maintained barrier suggest an improved functional outcome post stroke. The Clark’s score system assesses both general and focal deficits to monitor progression of general well-being and recovery, respectively [27]. Stroke induced significant and similar grades of general and focal deficits 1 day after stroke (Fig. 8a, b). All groups showed improved recovery at 3 days with overall well-being unaffected by the loss of HIF-1 signaling (Fig. 8a). However, Stroke-HIF-1 LoF mice had significantly lower focal deficit scores at 3 days post stroke compared to Stroke-Ctrl indicating better neurological function (Fig. 8b). Next neurobehavioural tests were used to evaluate sensorimotor function. These assessments were performed 1 day prior to surgery (baseline) and at 3 days reperfusion. Normal baseline (pre-surgery) sensorimotor performance was significantly impaired by ischemic insult in both groups (Fig. 8c–e). However, Stroke-HIF-1 LoF mice had much better locomotor activity as shown by the shorter times (up to 50 %) required to complete the latency test compared to Stroke-Ctrl animals (Fig. 8c). Similar results were obtained with the ladder rung test. Increased foot placement errors were observed in both groups but Stroke-HIF-1 LoF mice had fewer fore- and hindlimb faults compared to Stroke-Ctrl (Fig. 8d). As stroke-induced unilateral damage results in preferential turning in the ipsilateral direction, the corner test task was also performed. Although clear asymmetric turning behaviours was detected in both stroke groups compared to sham animals there was no difference in left turn preference between the stroke groups (Fig. 8e). As the corner test requires multiple sensory and motor coordination including vibrissae sensory, postural and limb biases [29], it is likely the animals need longer recovery times to show differences with this test. Overall, the HIF-1 LoF mice performed consistently better showing reduced neurological deficits.

## Discussion

The role of HIF-1 stabilization during stroke remains controversial [14, 36–39]. As accumulating evidence shows increased barrier permeability results in exaggerated lesion progression, better understanding of how HIF-1 and vascular function are entwined is warranted. This study provides convincing evidence that HIF-1 stabilization in brain pericytes negatively impacts stroke severity and progression. Preventing HIF-1 activation reduced infarction, improved neuronal survival and barrier stability, and enhanced neuronal function post ischemia. Blocking HIF-1 also reduced pericyte apoptosis particularly in peri-infarct areas resulting in improved vessel wall coverage, better barrier stability and enhanced protection of salvageable cells. Thus, our data highlights that protecting pericytes and maintaining barrier functionality during early stroke stages reduces brain damage and consequently improves stroke outcome.

Logically, as a well-described neuroprotective factor, boosting HIF-1 stabilization is an attractive treatment strategy for stroke [40, 41]. However detrimental effects in animal stroke models [36, 38, 39, 42, 43], and other injury paradigms such as traumatic brain injury [44], means global HIF-1 stabilization could exaggerate disease progression. Our study underlines this notion as loss of pericyte HIF-1 significantly reduced the degree of infarction and cerebral edema post stroke. The positive effects of HIF-1 LoF were mainly limited to peri-infarct areas suggesting severe damage in the ischemic core is largely irreversible but protection of salvageable peri-infarct cells occurs. This is an important observation as surviving neuronal peri-infarct populations have the ability to remap sensorimotor function of damaged areas, thereby aiding neurological recovery following stroke. Indeed, reduced neuronal degeneration and infarction aligned with clear improvements in sensory and motor function testing in the HIF-1 LoF mice. It seems unprecedented that such an outcome could result from manipulation of HIF-1 solely in a relatively small cell population that does not have direct neuronal connections. However we [12, 45] and others [19–22] have shown that the close interactions between cells of the NVU, and BBB in particular, means cell-specific alterations have high potential to modulate multiple cell types and thereby impact injury outcome. In line with this view, enhanced astrocyte activation observed in our HIF-1 LoF mice may also contribute to reduced injury as astrocyte aquaporin-4 expression facilitates reabsorption of vasogenic fluids from the extracellular space post stroke [31]. Clearly, their intimate contact with the endothelium gives pericytes an unprecedented ability to indirectly influence multiple cells and brain characteristics. Activation of HIF-1 signaling clearly compromises their functionality.

Vascular remodeling is mediated by a cascade of genes, many of which are HIF-1 targets. Although the cellular origin of these signals remains largely unclear, vascular remodeling was completely abrogated in LoF mice indicating that pericyte HIF-1 is a major regulator of this stroke-induced effect in vivo*.* Multiple HIF-1 downstream targets could be responsible however and it seems likely that concerted action is at work. A clear contender is VEGF, a potent inducer of vascular leak post stroke that is linked to pericyte-mediated vascular remodeling [46]. Another culprit could be matrix metalloproteinase-9 (MMP-9) since suppressing its secretion in pericytes prevented degradation of the vascular basal lamina post ischemia [47]. We consistently noted that ischemia induced brain levels of VEGF but surprisingly, no differences between Ctrl and HIF-1 LoF animals in serum or various brain regions were detected. Similarly, no differences in regional numbers of infiltrating cells were noted between the stroke mice. As it is highly likely that local changes initiated by pericytes at the vessel wall cannot be easily detected, more sophisticated techniques will be needed to gain better insight of these complex pathways. Regarding barrier integrity per se, interesting discrepancies between junctional protein levels in the ischemic core and peri-infarct regions were noted. In the ischemic core levels of Claudin-5, VE-cadherin and β-catenin were maintained in LoF animals whereas Stroke-Ctrl mice had elevated levels of these proteins in correlation with the increased permeability observed. As VE-cadherin and β-catenin induction aid vascular stabilization [48, 49] this mechanism is likely redundant in Stroke-HIF-1 LoF mice due to the better preserved barrier. On the other hand, frequently observed depletion of Occludin and ZO-1 [50, 51] was not recovered perhaps underlining the importance of the *bona fide* TJ protein Claudin-5. In the peri-infarct regions, absence of any differences between Ctrl and LoF expression levels at this acute stage is in line with a study by Knowland et al. [52]. It is noteworthy however, that TJ localization and vascular structure *per se* was considerably improved in the peri-infarct of LoF mice in agreement with reduced barrier permeability as seen by others [53, 54]. The correlation of pericyte coverage with BBB functionality establishes a causal link between increased pericyte survival and better outcome. Thus it seems HIF-1 LoF does not protect against the first hit (i.e. in the ischemic core) but reduces detrimental changes and ultimately salvages cells in peri-infarct regions. It could be interesting to evaluate whether pericyte HIF-1 modulates ischemia-induced expression of Claudin-1, a TJ protein shown to induce BBB permeability [10]. Although not investigated here it is also conceivable that HIF-1 may also play a role in transendothelial leakage.

Continuous interaction and communication of pericytes with the endothelium is critical for vascular stability and pericyte loss leads to uncontrolled angiogenesis and barrier leak [19, 20]. Indeed, oxidative and nitrosative stress during ischemia triggers pericyte apoptosis followed by a state of rigor resulting in long-lasting blood flow reduction and BBB breakdown [55]. Stroke-induced pericyte death and reduced coverage was clearly prevented by HIF-1 LoF. Being a well known double-edged sword, on the one hand protecting cells while on the other activating cell death mechanisms [2, 40], we conclude that severe stroke conditions per se induce negative HIF-1 effects in the pericyte compartment. Pericyte death may be mediated by HIF-1-induced p53 as elevated expression has been observed in pericytes of different origin under various injury conditions [56, 57]. However, evidence also suggests activated pericytes acquire an ability to become multipotent stem cells, differentiating into neuronal and microglial lineage cells post stroke [58]. As these stem cells might no longer express classic pericyte markers such as PDGFR-β or NG-2 rendering them undetectable, we cannot discount that HIF-1 driven de-differentiation of pericyte populations also occurs. A puzzling question is why pericytes are more sensitive to ischemic injury compared to endothelial cells or astrocytes, as we did not detect cell death in these peri-infarct populations. This data contrasts with that from in vitro models showing pericytes and astrocytes are more tolerant to injury conditions than endothelial cells [59, 60], although astrocytes were more hardy than pericytes. Perhaps these in vitro studies using single cell types miss pivotal paracrine signals that modify cellular responses in certain conditions. What those signals are remains elusive but recent studies by our group [61] and others [62] suggest that metabolic crosstalk may be critical. HIF-1 induces a multitude of target genes, so identifying the specific culprits will require considerably more in-depth investigation.

As pericyte HIF-1 induction clearly harmed brain vascular function and impairs functional recovery post stroke, preventing its induction could be a novel approach to reduce ischemic injury. However, this is a complicated goal since HIF-1 is a multifunctional transcription factor. Interestingly, HIF-1 stabilizing drugs are already being tested in phase 2 chronic kidney disease clinical trials [63] and hope for their use in stroke therapy is high. Our data suggests the use of global HIF-1 stabilizers to boost neuronal survival during brain injury must be carefully scrutinized as consequent barrier disturbance is clearly of high relevance. Indeed, the possibility that global HIF-1 stabilizers could worsen stroke outcome is not without precedence. A way around the problem would be to specifically target HIF-1 in pericytes and/or prevent loss of coverage. Some recent studies have provided hope in this direction as reviewed by Cheng et al. [64]. Alternatively, as pericyte coverage and BBB disruption go hand in hand, direct support of the endothelial barrier alone might reap significant benefits. Indeed, a BBB maintenance strategy could have strong potential to extend the temporal window and safety of current treatments [65]. Recent data suggests that supplementation of glutathione may be a potential candidate to achieve this aim [66, 67]. An obvious advantage of such therapies would be straightforward application via the circulatory system.

Although our findings demonstrate the importance of pericyte HIF-1 stabilization during early stroke, some caveats need to be addressed in future studies. Firstly, we cannot rule out potential contribution and/or beneficial effects of smooth muscle cell HIF-1 deletion in this study, as the SMMHC promoter is not restricted to pericytes. We are also mindful that distinct differences in vascular pathophysiology along arterial, capillary, and venous segments of the cerebral vasculature occur during stroke. However other studies also confirm the SMMHC-CreER^T2^ system localizes mainly to pericytes in the capillary bed [68, 69] and that stroke-induced BBB permeability is least observed in arteries and most pronounced in capillaries [70]. We have not yet examined the outcome of pericyte HIF-1 LoF during more chronic phases of stroke. Cellular crosstalk at the BBB, and NVU in general, is mediated at least in part by exchange of soluble factors [2]. In this regard HIF-1 regulates secretion of a plethora of growth factors that have biphasic roles during stroke progression i.e. beneficial during the acute phase but deleterious during the chronic phase, and vice versa [71, 72]. Therefore, future studies that carefully examine outcome during chronic stroke phases are also needed.

In summary pericyte HIF-1 stabilization plays a key role in acute stroke severity and outcome. Looking forward, understanding if these beneficial effects persist long-term will provide valuable translational information. Given the ubiquity of HIF-1 signaling during pathological processes, conclusions and concepts derived from this study could be very relevant in other brain diseases such as traumatic brain injury, multiple sclerosis and age-related vascular dysfunction.

## Electronic supplementary material

Below is the link to the electronic supplementary material.

(PDF 15,613 kb)

(DOCX 17 kb)

(DOCX 14 kb)
