# Supplementary material for: Pericyte hypoxia-inducible factor-1 (HIF-1) drives blood-brain barrier disruption and impacts acute ischemic stroke outcome
Source: Angiogenesis. 2021 May 27;24(4):823–42. doi: 10.1007/s10456-021-09796-4 (PMC8487886; doi:10.1007/s10456-021-09796-4)
Supplement: Supplementary file 1 — (PDF 15,613 kb) [file 10456_2021_9796_MOESM1_ESM.pdf]

Supplementary figures

Supplementary Fig. 1

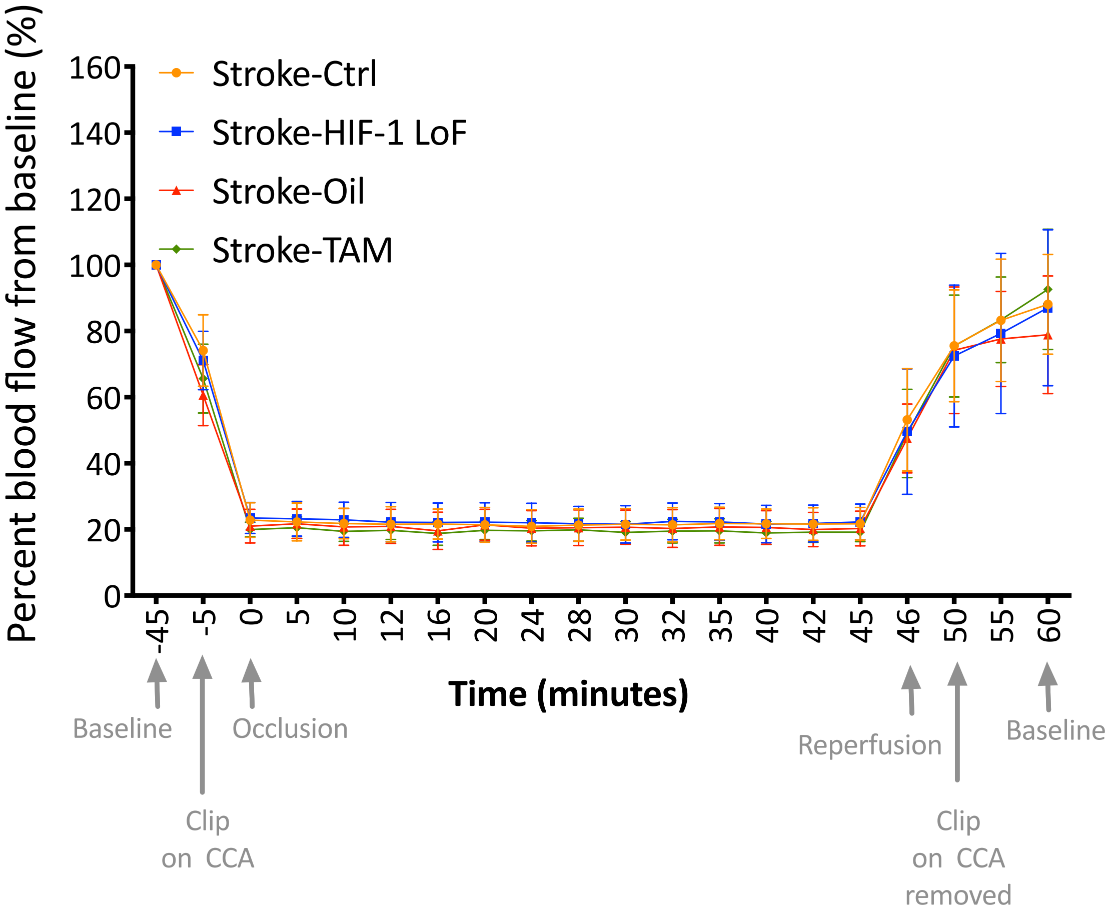

Supplementary Fig. 2

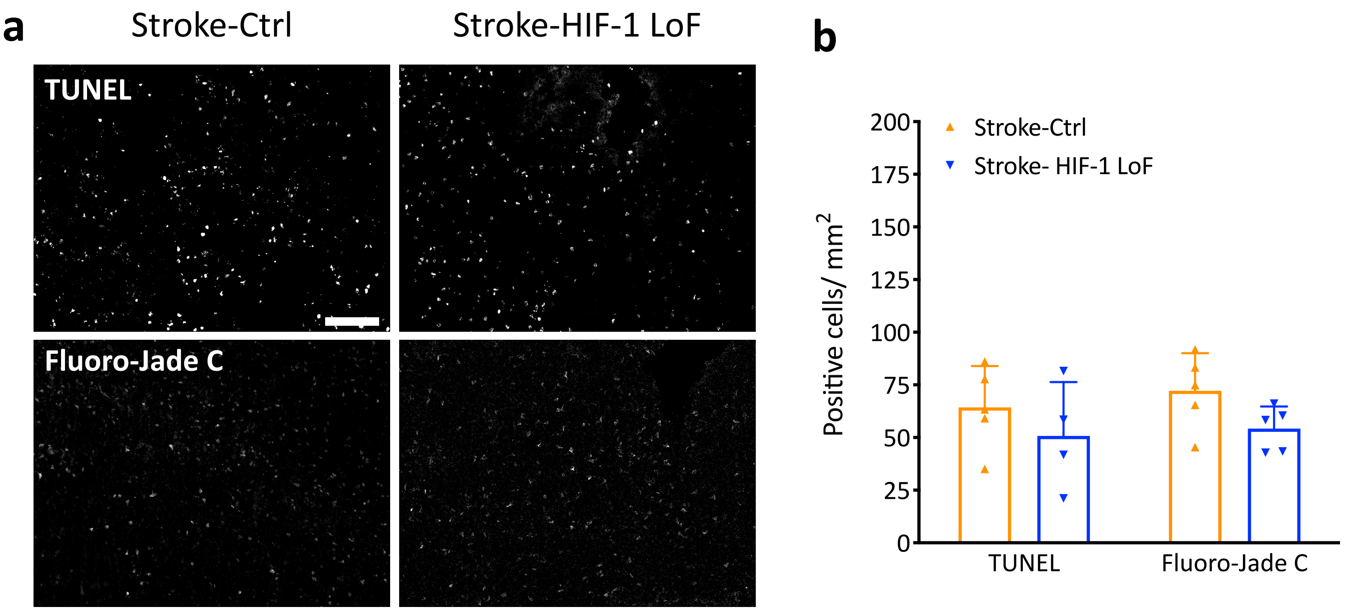

Supplementary Fig. 3

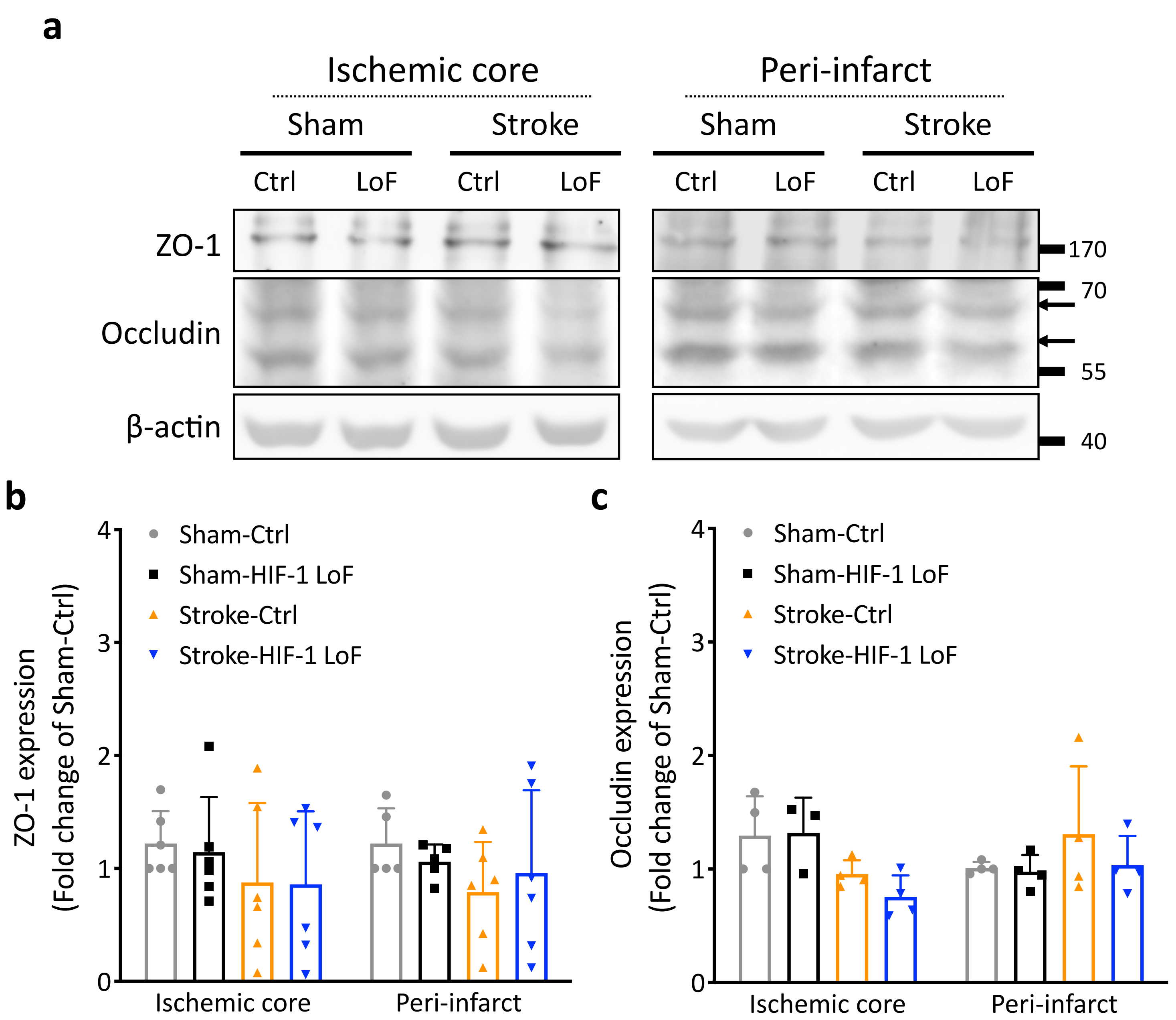

Supplementary Fig. 4

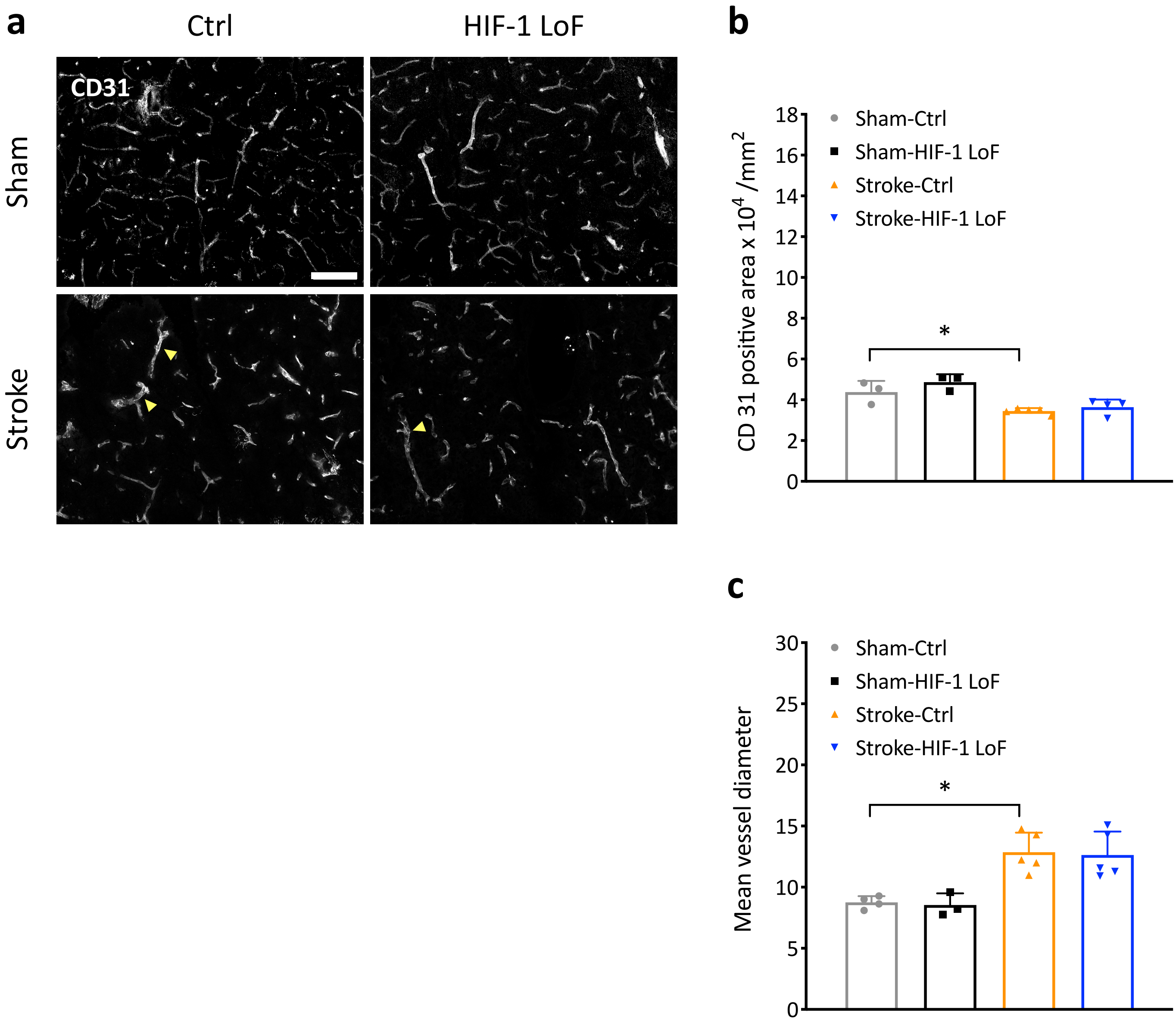

Supplementary Fig. 5

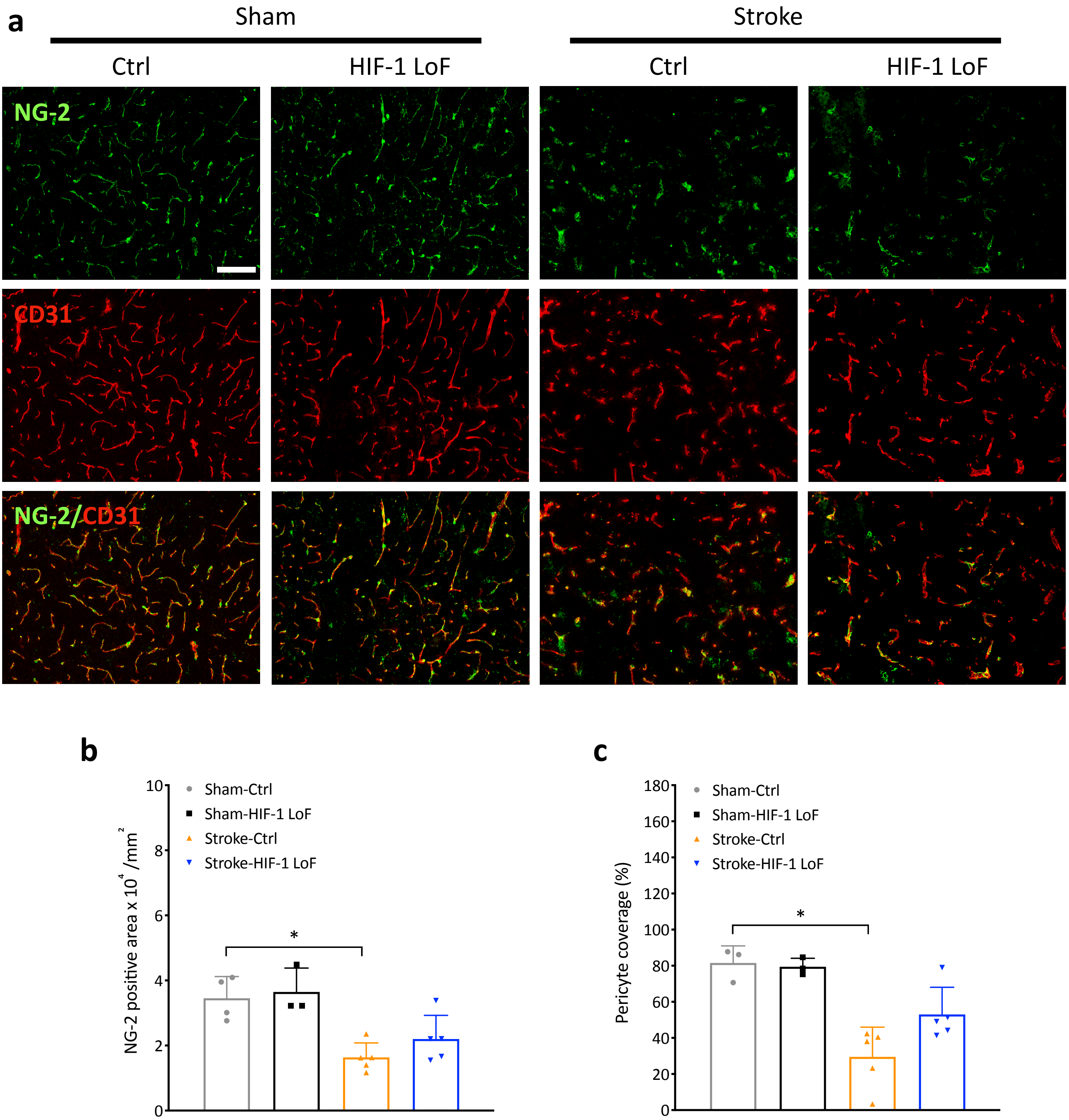

Supplementary Fig. 5

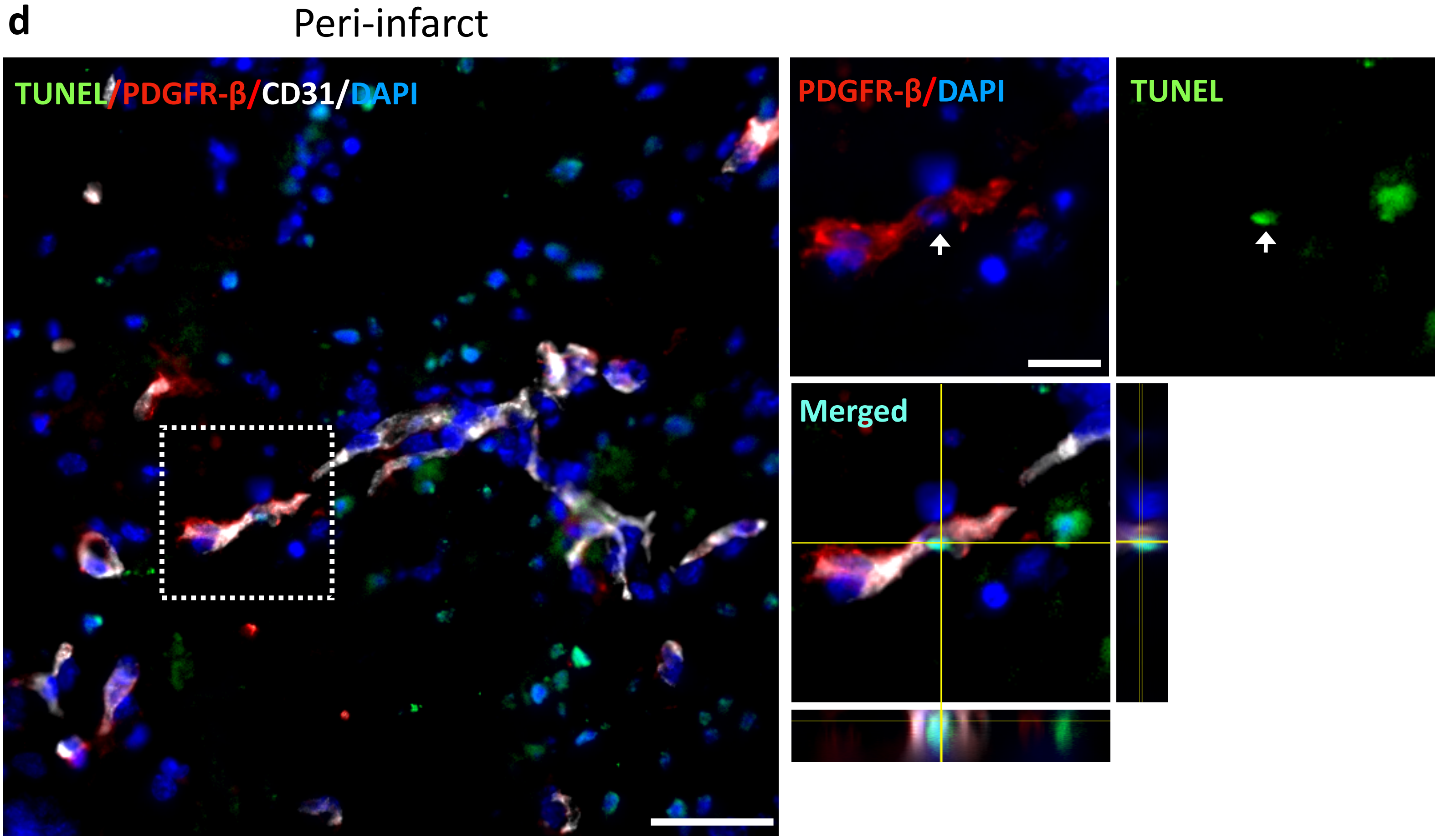

Supplementary Fig. 6

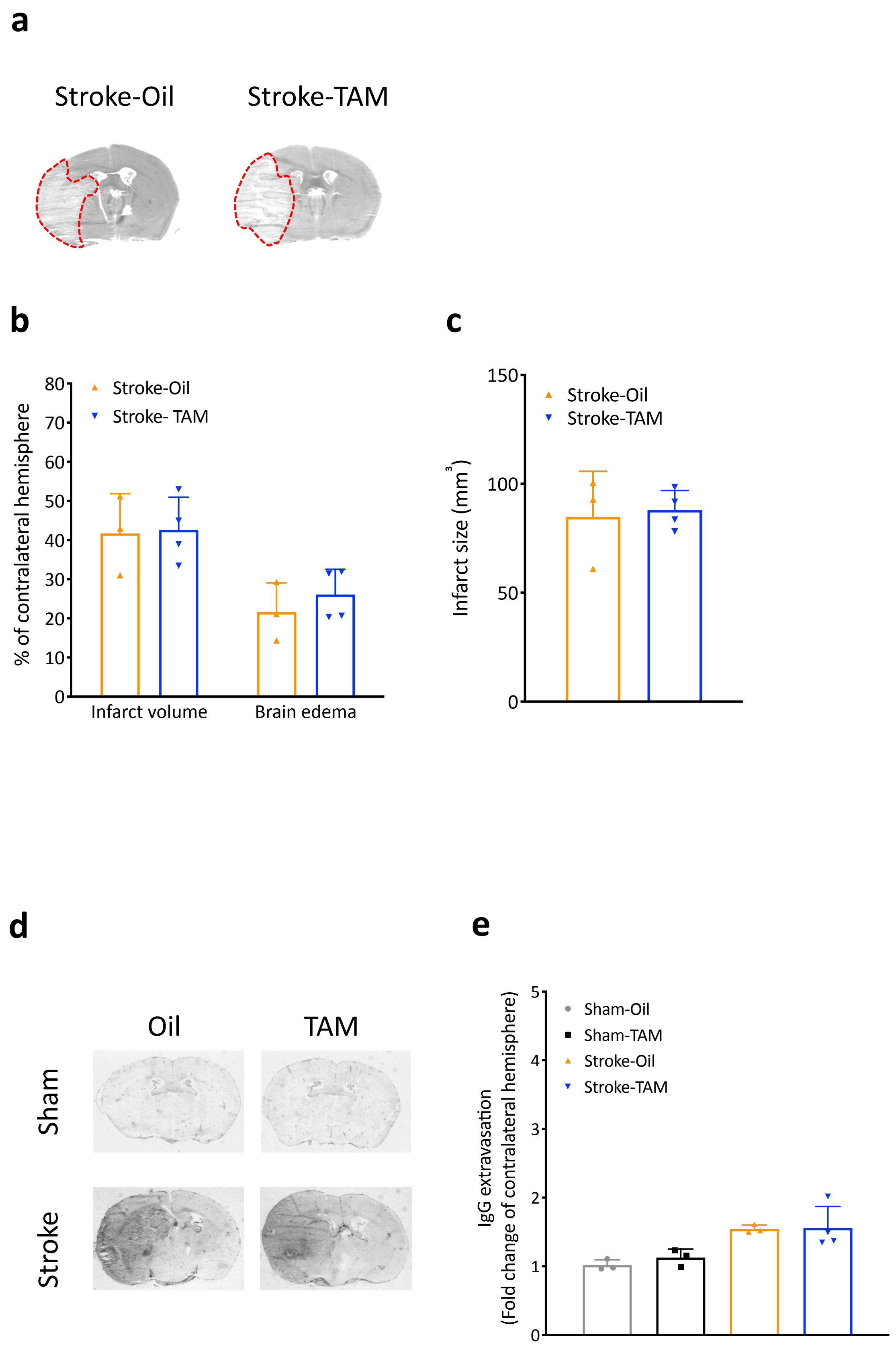

Supplementary Fig. 6

f

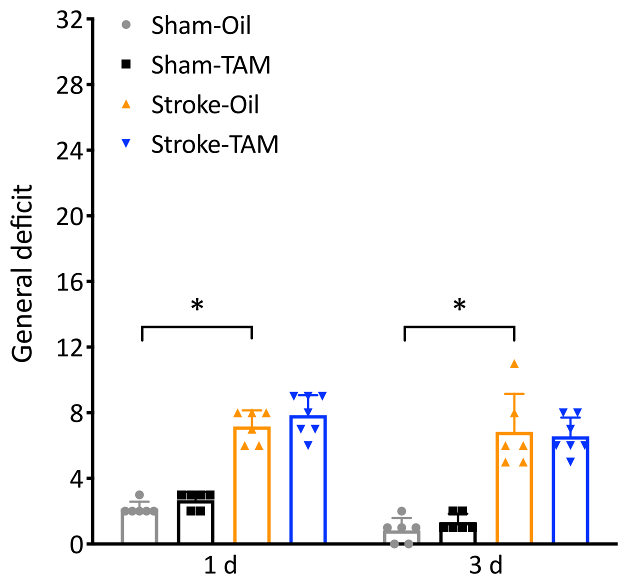

g

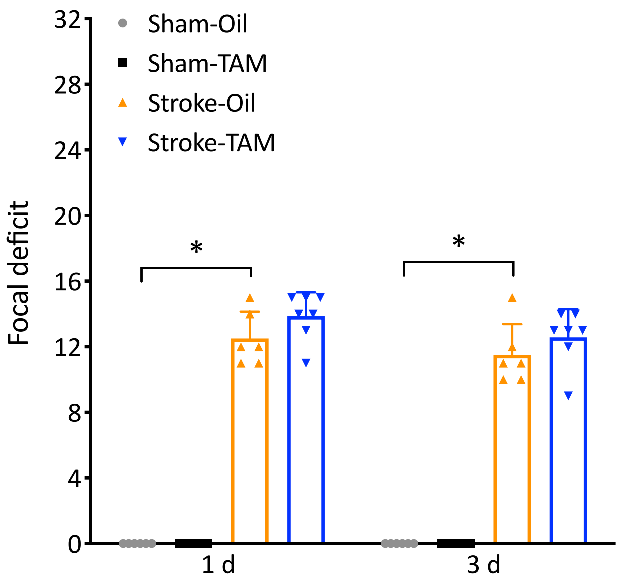

h

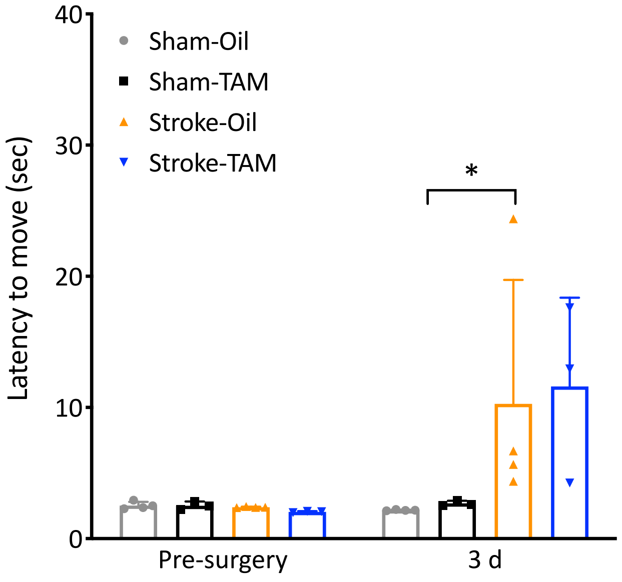

Supplementary Table S1

List of PCR and qRT-PCR primers used in this study

List of PCR primers used in this study

Primer pair	Forward	Reverse
**Floxed HIF-1α**	5'-GCAGTTAAGAGCACTAGTTG-3'	5'-GGAGCTATCTCTCTAGACC-3'
**SMMHC**	5'-TGACCCCATCTCTTCACTCC-3'	5'-AACTCCACGACCACCTCATC-3'
**SMMHC-CreER^{T2}**	5'-AGTCCCTCACATCCTCAGGTT-3'	5'-AACTCCACGACCACCTCATC-3'
**Truncated HIF-1α**	5'-GGGATGAAAACATCTGCTTTGGA-3'	5'-TGTGTTGGGGCAGTACTGG-3'

List of qRT-PCR primers used in this study

Primer pair	Forward	Reverse
**HIF-1α exon2**	5'-CGGCGAAGCAAAGAGTCTGAAG-3'	5'GATGGTGAGCCTCATAACAGAAGC-3'
**Glut-1**	5'-GGGCATGATTGGTTCCTTCTC-3'	5'-CAGGTTCA TCATCAGCATGGA-3'
**VEGF**	5'-CGCAAGAAATCCCGGTTTAA-3'	5'-CAAATGCTTTCTCCGCTCTAA-3'
**β-actin**	5'-CTGGCTCTAGCACCATGAAG-3'	5'-GCCACCGATCCACACAGAGT-3'
