# Supplementary material for: Pericyte hypoxia-inducible factor-1 (HIF-1) drives blood-brain barrier disruption and impacts acute ischemic stroke outcome
Source: Angiogenesis. 2021 May 27;24(4):823–42. doi: 10.1007/s10456-021-09796-4 (PMC8487886; doi:10.1007/s10456-021-09796-4)
Supplement: Supplementary file 2 — (DOCX 17 kb) [file 10456_2021_9796_MOESM2_ESM.docx]

**SUPPLEMENTARY MATERIAL AND METHODS**

*Microvessel and primary pericyte isolation*

Primary brain pericytes were isolated from 10-12 weeks old SMMHC–CreER^T2^; HIF-1α^flox/flox^ mice according to Milner *et al.*[73] with slight modifications. After removing meninges, cortices were homogenized and digested with 30 U/ml papain and 40μg DNase I for 45 min at 37°C then dissociated using a 19G needle. Microvessels were then extracted by centrifugation (10 min at 1360 g at 4°C) using 22% BSA and plated on rat tail collagen coated dishes and cultured in endothelial cell growth medium (Milipore, USA). Adherent pericytes were subsequently passaged and maintained in DMEM supplemented with 20% FBS, 50μg/ml gentamycin sulphate. For all experiments, pericytes were used at passage 1 after purity of the cultures was confirmed ≥95% by immunostaining for standard cell markers NG-2 and PDGFR-β.

*PCR and Quantitative real-time PCR (qRT-PCR)*

Deletion of the floxed HIF-1α exon2 was confirmed using genomic DNA isolated from cortices of tamoxifen/oil injected animals. The PCR analysis was performed using primers listed in Supplementary Table S1. The PCR products were visualized after being run on 1.5 % agarose gels at 50 V for 60 min.

To further confirm loss of HIF-1 functionality, pericytes were isolated from SMMHC-CreER^T2^; HIF-1α^flox/flox^ mice. Cultured primary mouse pericytes were treated with oil or tamoxifen (2 µM) during 48 h normoxia/hypoxia (1% O_2_). Total RNA was then isolated from the pericytes using PureLink® RNA Mini Kit (Invitrogen, USA) and 1μg reverse transcribed using the ImProm-II ReverseTranscriptase kit (Promega, USA). qRT-PCR was performed with an ABI 7500 Fast Real-Time PCR System using Power Sybr® Green PCR Master Mix (Applied Biosystems, UK). Primers for HIF-1α exon 2, Glut-1, VEGF and β-actin are presented in Supplementary Table S1. Fold changes were calculated using the comparative ΔΔCt method and normalized to β-actin.
