# Supplementary material for: Pericyte hypoxia-inducible factor-1 (HIF-1) drives blood-brain barrier disruption and impacts acute ischemic stroke outcome
Source: Angiogenesis. 2021 May 27;24(4):823–42. doi: 10.1007/s10456-021-09796-4 (PMC8487886; doi:10.1007/s10456-021-09796-4)
Supplement: Supplementary file 3 — (DOCX 14 kb) [file 10456_2021_9796_MOESM3_ESM.docx]

**Supplementary figure legends**

**Supplementary Figure 1: No effect of HIF-1 LoF/tamoxifen on cerebral blood flow (CBF) during tMCAo/reperfusion.**

Percentage cortical CBF of SMMHC-CreER^T2^; HIF-1α^flox/flox^ mouse line (Stroke-Ctrl/Stroke-HIF-1 LoF) and SMMHC-CreER^T2^ mouse line (Stroke-Oil/Stroke-TAM) was measured by laser doppler flowmetry throughout tMCAo intervention. Percentage values were calculated by normalizing to baseline blood flow. Significant and steady reduction of CBF occurred when MCA was occluded for 45 min in all groups. Recovery to near baseline occurred after filament withdrawal. CCA: common carotid artery. Mean ± SD, n = 16-18

**Supplementary Figure 2: Pericyte HIF-1 LoF does not alter apoptosis or neuronal degeneration in the ischemic core.**

**a,b** Representative images **(a)** and quantification **(b)** of TUNEL and Fluoro-Jade C staining in the ischemic core. Scale bar = 100 µm. No difference was seen between the stroke groups. Unpaired t-test compared to Stroke-Ctrl. Mean ± SD. n = 4-5.

**Supplementary Figure 3: ZO-1 and Occludin levels are unchanged in the ischemic core and peri-infarct area at 3 days reperfusion.**

**a-c** Representative Western blot **(a)** and quantification of ZO-1 **(b)** and Occludin **(c)** expression in the ischemic core and peri-infarct at 3 days post-stroke. β-actin is the loading control. Two-way ANOVA. Mean ± SD. n = 4-5.

**Supplementary Figure 4: Vascular patterning in the ischemic core is unaffected by pericyte HIF-1 loss of function.**

**a** Representative images of CD31 staining in the different groups with arrowheads marking vasodilation. **b,c** Histogram of CD31-positive areas **(b)** and mean vessel diameter **(c)**. Scale bar = 100 µm. Two-way ANOVA. Mean ± SD. n = 4-5.

**Supplementary Figure 5: HIF-1 LoF marginally improves ischemic core pericyte coverage.**

**a** Representative images of NG-2 (green) and CD31 (red) staining in the ischemic core in all animal groups at 3 days after stroke. Scale bar = 100 µm. **b,c** Histograms show NG-2 positive area **(b)** and pericyte coverage **(c)**. Two-way ANOVA. *P<0.05. Mean ± SD. n = 4-5. **d** Representative image showing pericyte death in Stroke-Ctrl peri-infarct. Section is co-stained with TUNEL (green), PDGFR-β (red) and CD31 (white) and counterstained with DAPI (blue). The images on the right are 1.6X magnifications of the boxed region with TUNEL/NG-2 double positive cells within a vessel highlighted with arrows. Merged orthogonal views of horizontal and vertical Z-stack images confirm TUNEL localizes to the nucleus of a PDGFR-β positive pericyte. Scale bar = 50 µm.

**Supplementary Figure 6: Tamoxifen has no effect on stroke outcome, barrier permeability or functional testing in SMMHC-CreER^T2^ control mouse line.**

**a** Representative images of H&E stained coronal brain sections with infarct area delineated with a red dotted line. **b,c** Histograms of infarct volume, brain edema **(b)** and infarct size **(c)**. **d,e** Representative images **(d)** and quantification **(f)** of IgG extravasation, and thus vessel permeability, 3 days post stroke. **f,g** Clark’s score with general deficits **(f)** and focal deficits **(g)** at 1 day and 3 days post-stroke. **h** Results of latency to move neurobehavioural test. Two-way ANOVA. *P<0.05. Mean ± SD. n = 4-5.

**Supplementary Table S1: List of PCR and qRT-PCR primers used in the study.**

Table shows forward and reverse primer sequences used for gene amplification.
